# Concomitant medication use and clinical outcome in hepatocellular carcinoma treated with immune-based therapy: a multicenter analysis

**DOI:** 10.3389/fimmu.2025.1680015

**Published:** 2025-11-28

**Authors:** Jun-Zhe Yi, Jie Xu, Jiang-Zheng Zeng, Yu-Jia Song, Yu-Nan Zhang, Cheng-Mou Huang, Lei Li, Ke Ding, Zhi-Jian Zhu, Dao-Zu Yuan, Yu-Wen Fan, Shang-Fu Ning, Xin Liu, Jian-Zhong Zhang, Yong-Fei You, Qi Zhou, Hao-Jie Song, Gen-Jun Tan, Xin-Tong Wu, Rong-Ping Guo, Min-Shan Chen, Ning Lyu, Ming Zhao

**Affiliations:** 1 Department of Minimally Invasive Interventional Therapy, Liver Cancer Study and Service Group, State Key Laboratory of Oncology in South China, Guangdong Provincial Clinical Research Center for Cancer, Sun Yat-sen University Cancer Center, Guangzhou, China; 2 Department of Medical Oncology, The First Affiliated Hospital of Hainan Medical University, Haikou, China; 3 Department of Oncology, Guangzhou Chest Hospital, Guangzhou, China; 4 Department of Oncology, Jiangmen Affiliated Hospital of Chinese Medicine, Jinan University, Jiangmen, China; 5 Department of Medical Oncology, Ganzhou Cancer Hospital, Ganzhou, China; 6 Department of Oncology, Chengdu Seventh People’s Hospital, Chengdu, China; 7 Department of Oncology, Chenzhou Municipal Hospital of Traditional Chinese Medicine, Chenzhou, China; 8 Department of Medical Oncology, Wuchuan People’s Hospital, Wuchuan, China; 9 Department of Oncology, Yantai Hospital of Traditional Chinese Medicine, Yantai, China; 10 Department of Oncology, Yunan County People’s Hospital, Yunan, China; 11 Department of Oncology, The First Hospital of Nanchang, Nanchang, China; 12 Department of Infectious Disease, Guangyuan Central Hospital, GuangYuan, China; 13 Department of Oncology, Boai Hospital of Zhongshan, Zhongshan, China; 14 Department of Liver Surgery, State Key Laboratory of Oncology in South China, Guangdong Provincial Clinical Research Center for Cancer, Sun Yat-sen University Cancer Center, Guangzhou, China

**Keywords:** hepatocellular carcinoma, immunotherapy, concomitant medication, clinical outcomes, treatment-related adverse events

## Abstract

**Introduction:**

Immune checkpoint inhibitors (ICIs) have been increasingly used in hepatocellular carcinoma (HCC). Despite emerging evidence indicating that concomitant medications might impact clinical outcomes, their association with ICI efficacy is undefined in HCC.

**Methods:**

The multicenter cohort included 851 HCC patients receiving ICIs between January 2018 and December 2022 in 13 institutions. Concomitant medications given within 30 days before or after the initiation of ICIs were evaluated in association with survival, tumor response, and treatment-related adverse event (TRAE) occurrence. Concomitant medications administered within 30 days before or after the initiation of ICI therapy were identified and used to categorize patients into user and non-user groups for each medication class. The primary outcomes were overall survival (OS), and secondary outcomes included progression-free survival (PFS), time to progression (TTP), tumor response, and TRAEs. Tumor response was evaluated based on RECIST 1.1. Multivariable Cox and logistic regression models were used to adjust for confounding variables.

**Results:**

The median OS (13.6 vs. 20.7 months; P = 0.006) and PFS (6.6 vs. 8.9 months; P = 0.002) were significantly reduced for antibiotic users compared to non-users, while other drugs did not show an impact on patient outcomes. In multivariable analysis, antibiotic use predicted worse survival outcomes (OS: HR = 1.88, 95% CI, 1.14–3.11; P = 0.014; PFS: HR = 1.60, 95% CI, 1.20–2.13; P = 0.001). Patients who underwent glucocorticoids for early TRAE management achieved longer OS than those for prophylactic use (OS: Not reached vs. 20.3 months; HR = 0.25, 95% CI, 0.10–0.65; P = 0.042). Concomitant use of medications such as antibiotics, H2RAs, and glucocorticoids was associated with increased incidence of specific TRAEs, particularly involving hematologic, hepatic, and endocrine systems.

**Conclusion:**

This study identified antibiotic use as a negative prognostic factor in HCC treated with ICIs, while glucocorticoid for early TRAEs may indicate improved survival benefits. Concomitant medication may affect the occurrence of TRAEs and warrants careful consideration during ICI treatment.

## Highlights

- Concomitant antibiotic use may diminish the survival outcomes of immune-based therapy for HCC.
- Concomitant glucocorticoid use did not attenuate immunotherapy efficacy in HCC and its use for early TRAEs may indicate improved survival in HCC.
- Concomitant medications were associated with the altered spectrum of adverse events related to immune-based therapy.

## Introduction

Hepatocellular carcinoma (HCC) typically presents at an advanced stage, resulting in a dismal prognosis and ranking as the third leading cause of cancer-related mortality worldwide (1, 2). Since 2020, the advent of immune-based therapy has reshaped the treatment landscape of unresectable/advanced HCC (3). Various combination approaches, incorporating immune checkpoint inhibitors (ICIs) targeting programmed cell death-1 receptor/ligand (PD-1/PD-L1), in conjunction with anti-angiogenic agents, cytotoxic T-cell lymphocyte antigen-4 blockade, or tyrosine kinase inhibitors (TKIs), have demonstrated prolonged survival outcomes in numerous global phase 3 trials (4–9). Recent studies have also shown encouraging results for combining immune-based therapies with locoregional approaches in patients with unresectable HCC (10). During ICI treatment, HCC patients often receive concomitant medications to manage cancer-related symptoms or comorbidities (e.g., chronic inflammation, metabolic syndrome, and cachexia) (11, 12). These medications may exert immune-modulatory effects within the tumor immune microenvironment (TIME) (13, 14), raising concerns regarding their potential adverse impact on the efficacy of immune-based therapies.

Among the concomitant medications, antibiotics have garnered particular attention due to their profound impact on gut microbiota, which plays a pivotal role in shaping antitumor immune responses (13, 15–17). By disrupting microbial diversity and intestinal homeostasis, antibiotics may impair ICI efficacy and increase the risk of treatment-related toxicity (13, 17). Glucocorticoids are typically employed to manage immune-related adverse events during ICI therapy. Their well-established immunosuppressive effects—such as reducing naïve T cell proliferation and promoting regulatory T cell differentiation—may weaken antitumor immunity and reduce ICI effectiveness (18, 19). Gastric acid suppressants, including proton pump inhibitors (PPIs) and histamine-2-receptor antagonists (H2RAs), are frequently administered to alleviate gastroduodenal ulcers and gastroesophageal reflux in HCC patients (20, 21). These medications have been shown to significantly alter the composition of gut microbiota through modulating stomach acidity and exerting direct effects on microbial populations (22–24). Other commonly prescribed medications such as antidiabetic and antihypertensive drugs, nonsteroidal anti-inflammatory drugs (NSAIDs), and opioids have been reported to modulate immune status within the TIME (25–27).

Currently, although accumulating evidence suggests the association between multiple common medications and immunotherapy outcomes in several malignancies (14, 28–30), their therapeutic implications in HCC remain unclear. Antibiotics have been preliminarily investigated in HCC patients undergoing ICIs. However, conclusive findings have been elusive due to the inconsistent data (31–33), and the therapeutic effects of antibiotics are often assessed independently, neglecting potential interactions with other commonly used medications. Moreover, evidence regarding the impact of several concomitant medications, including antidiabetic/antihypertensive agents, NSAIDs, and opioids, in the HCC population is lacking.

To address these knowledge gaps, we conducted the first nationwide cohort study to comprehensively evaluate the impact of multiple concomitant medications on clinical efficacy and safety of immune-based therapy in HCC patients. By integrating large-scale, real-world data from multiple institutions, our study provides clinical evidence on whether commonly prescribed medications influence ICI treatment outcomes. These findings may offer actionable insights to support risk–benefit assessments and guide more individualized treatment strategies for patients with HCC.

## Material and methods

### Study design

This retrospective multicenter cohort study included HCC patients who received ICI therapy either as monotherapy or combination regimens in 13 Chinese institutions from January 2018 to December 2022 (**Supplementary Table S1**). We systematically reviewed medical records and extracted data related to radiological findings, laboratory examinations, and follow-up information. Specifically, baseline patient characteristics (e.g., demographic data, general condition, laboratory and radiological results), treatment information, and post-treatment follow-up imaging records were obtained from the electronic medical record system. The survival status of patients was confirmed through regular telephone follow-ups. Eligible patients had to fulfill the following inclusion criteria: (1) measurable lesions per Response Evaluation Criteria in Solid Tumors version 1.1 (RECIST 1.1); (2) unresectable HCC that was not amenable to or had progressed after surgery or ablative therapy based on the Barcelona Clinic Liver Cancer (BCLC) staging system; (3) Eastern Cooperative Oncology Group performance status (ECOG PS) of 0–2; (4) adequate organ and marrow function; and (5) adequate liver function (Child-Pugh class A or B7). Patients were excluded if they met any of the following criteria: (1) absence of measurable intrahepatic lesions; (2) lack of baseline imaging or laboratory data; (3) history of secondary malignancies; or (4) loss to follow-up.

### Treatment

Patients with HCC received PD-1 inhibitors (pembrolizumab, tislelizumab, sintilimab) or PD-L1 inhibitors (atezolizumab, durvalumab), either as monotherapy or in combination with bevacizumab (anti-angiogenesis agent) or lenvatinib (TKIs). The specific regimens and their corresponding proportions are detailed in **Supplementary Table S2**. HCC patients received PD-1/PD-L1 inhibitors alone or in combination with bevacizumab (anti-angiogenesis) or lenvatinib (TKIs), which was continued until disease progression or the occurrence of unacceptable toxicity. Concomitant medication exposure was defined as use within 30 days before or after the ICI initiation, consistent with criteria used in previous literature (30, 34). Patients who received the medication during this peri-treatment period were classified as exposed, irrespective of whether the medication was continued thereafter. Concomitant medications with potential immunomodulatory effects and a prevalence of ≥ 3% were screened and evaluated, including gastric acid suppressants (PPIs or H2RAs), antibiotics, glucocorticoids (defined as dose ≥ 10mg per day of a prednisone equivalent, with a minimum 24 hours of dosing) (29), NSAIDs, insulins, calcium channel blockers (CCBs), and opioids. For each medication category, the duration and timing of exposure relative to ICI initiation were collected and summarized in **Supplementary Table S3**. The initiation of corticosteroid therapy for immune-related adverse events (irAEs) in this study followed institutional protocols aligned with international clinical guidelines (35). Corticosteroids were administered for grade ≥ 2 irAEs, including but not limited to dermatologic reactions, immune-mediated hepatitis, colitis, and pneumonitis. The standard regimen typically consisted of oral or intravenous prednisone (or equivalent) at an initial dose of 0.5–1.0 mg/kg/day, followed by gradual tapering over several weeks based on clinical response. The decision to initiate corticosteroid treatment, as well as dosing and tapering schedule, was made at the discretion of the treating physician in accordance with established irAE management recommendations.

### Follow-up

Radiological examinations by magnetic resonance imaging or computerized tomography were performed before ICI initiation and approximately every 8–12 weeks thereafter. The primary outcome was overall survival (OS), and secondary outcomes included progression-free survival (PFS), time to progression (TTP), tumor response, and treatment-related adverse events (TRAEs). OS was defined as the time from the initiation of immunotherapy to any-cause death. PFS was defined as the time from the initiation of immunotherapy to the date of disease progression or any-cause death. TTP was defined as the time from the initiation of immunotherapy to the date of disease progression. Disease progression was determined based on either radiological response per RECIST 1.1 criteria (e.g., tumor enlargement or new lesion development) or clinical deterioration, including worsening liver function (e.g., change in Child-Pugh class) or declining performance status (e.g., ECOG ≥ 2). Tumor response was assessed using radiological imaging with RECIST 1.1 criteria, and evaluated through two complementary approaches (tumor response at the first imaging follow-up and tumor response based on best overall response [BOR]). The first imaging follow-up, typically conducted 8–12 weeks after initiation of immunotherapy, was used to assess early treatment response. BOR was defined as the most favorable radiological outcome from treatment initiation until radiological progression, treatment discontinuation, or the last available imaging. TRAEs were identified from clinical notes, laboratory and radiographic evidence, and recorded according to the Common Terminology Criteria for Adverse Events (CTCAE 5.0). The cutoff date for follow-up was November 30, 2023.

### Statistical analysis

Descriptive statistics were reported as frequency and proportions for categorical variables, and medians with ranges for continuous variables. Associations between concomitant medication and patient characteristics were assessed using the χ^2^-test. Survival outcomes including OS and PFS were analyzed employing the Kaplan-Meier method and compared using the log-rank test. Univariable and multivariable Cox proportional hazards models were applied to assess prognostic factors of survival outcomes. Variables with a P-value < 0.1 in univariable Cox model were included in multivariable analyses to calculate hazard ratios (HRs) and 95% confidence intervals (CIs). Besides, logistic regression models were utilized to establish the odds ratio (ORs) for tumor response and TRAEs. The χ^2^-test was used to compare the baseline characteristics between concomitant medication users and non-users and evaluate the association between concomitant medications and specific TRAEs. To minimize the immortal time bias, further clinical outcomes analyses were performed after applying a 30-day landmark selection, including only patients with a minimum treatment duration of 30 days. This strategy aimed to exclude early deaths unlikely related to ICI efficacy, thereby allowing a more accurate assessment of treatment-related survival outcomes. Additionally, we calculated the E-value to evaluate the robustness of our results against potential uncontrolled confounders (36). P-value < 0.05 was considered statistically significant. Statistical analyses were conducted using SPSS 26.0 and GraphPad Prism 9.0.

## Results

### Patient characteristics

In total, 851 consecutive HCC patients who received immune-based therapy were included in this study. Baseline characteristics and concomitant medication use were summarized in **Table 1**. The median age was 55 years (range, 18–89), and the most common underlying cause of liver disease was hepatitis B virus (HBV) infection (81.3%). A total of 562 patients (66.0%) presented with ≥ 3 intrahepatic tumor lesions. Macrovascular invasion and/or extrahepatic spread were observed in 574 (67.5%) individuals. Regarding the immunotherapy regimens, 316 patients (37.1%) received single PD-1/PD-L1 inhibitors, and 535 patients (62.9%) underwent combination therapy. The baseline characteristics of patients receiving each class of concomitant medication were compared with those of non-users and were summarized in **Supplementary Table S4**. Overall, these comparisons revealed no significant differences in key demographic and clinical variables, indicating that the groups were generally well-balanced at baseline.

**Table 1:** Patient characteristics.

	N = 851 (%)
Age, years; median (range)	55 (18–89)
Gender	
Male	753 (88.5)
Female	98 (11.5)
Etiology	
HBV	692 (81.3)
HCV	24 (2.8)
Other or unknown	158 (18.7)
ECOG PS	
0	286 (33.6)
≥ 1	565 (66.4)
Child-Pugh class	
A	678 (79.7)
B	173 (20.3)
BCLC stage	
A	34 (4.0)
B	229 (26.9)
C	588 (69.1)
Serum AFP level ≥ 400 ng/mL	
≥400:	401 (47.1)
<400:	450 (52.9)
Tumor diameter, cm	
> 10	199 (23.4)
≤ 10	652 (76.6)
Tumor number	
1–3	289 (33.9)
> 3	562 (66.0)
Macrovascular invasion	
Presence:	370 (43.5)
Absence:	481 (56.5)
Extrahepatic spread	
Presence:	348 (40.9)
Absence:	503 (59.1)
Macrovascular invasion and/or extrahepatic spread	
Presence:	574 (67.5)
Absence:	277 (32.5)
Treatment line of immunotherapy	
First-line	468 (55.0)
Second or subsequent line^#^	383 (45.0)
Type of systemic agent	
Anti-PD-1/PD-L1 monotherapy	316 (37.1)
Anti-PD-1/PD-L1 + lenvatinib	315 (37.0)
Anti-PD-1/PD-L1 + bevacizumab	220 (25.9)
Prior treatment for HCC	
Resection	268 (31.5)
Ablation	111 (13.0)
Trans-arterial interventional therapy	383 (45.0)
Tyrosine kinase inhibitor	347 (40.8)
Concomitant medications	
Antibiotic (yes)	78 (9.2)
Proton pump inhibitor (yes)	202 (23.7)
Histamine-2-receptor antagonist (yes)	50 (5.9)
Glucocorticoid (yes)	49 (5.8)
Nonsteroidal anti-inflammatory drug (yes)	99 (11.6)
Calcium channel blocker (yes)	46 (5.4)
Insulin (yes)	31 (3.6)
Opioid (yes)	98 (11.5)
Number of concomitant medication	
0	490 (57.6)
1	180 (21.2)
2	105 (12.3)
≥ 3	76 (8.9)

Data are shown as N (%), unless otherwise indicated.

AFP, α-fetoprotein; BCLC, Barcelona Clinic Liver Cancer; ECOG PS, Eastern Cooperative Oncology Group performance status; HBV, hepatitis B virus; HCV, hepatitis C virus. ^#^refers to systemic treatment administered following either progression on prior systemic therapy or locoregional interventions.

### Relationship between concomitant medications and immunotherapy efficacy

During a median follow-up of 25.7 months (95% CI, 23.6–27.2 months), 569 patients (66.9%) experienced disease progression and 434 patients (51.0%) died. The median OS of the total cohort was 19.4 months (95% CI, 17.5–23.9), and the median PFS was 8.8 months (95% CI, 7.8–9.8). Based on the radiographic response, there were 149 patients with complete or partial response (17.5%), 515 patients with stable disease (60.5%), and 187 patients with progressive disease (22.0%). At the data cutoff, ICI-based treatment was discontinued in 69.8% of patients. The median number of ICI cycles and median duration of ICI therapy were 4.0 (interquartile range [IQR], 2.0–9.0) and 5.0 months (IQR, 2.2–10.8), and the median duration of TKI exposure was 5.2 months (IQR, 2.4–10.7).

### Antibiotic use is associated with worse survival outcomes

**Figure 1:**
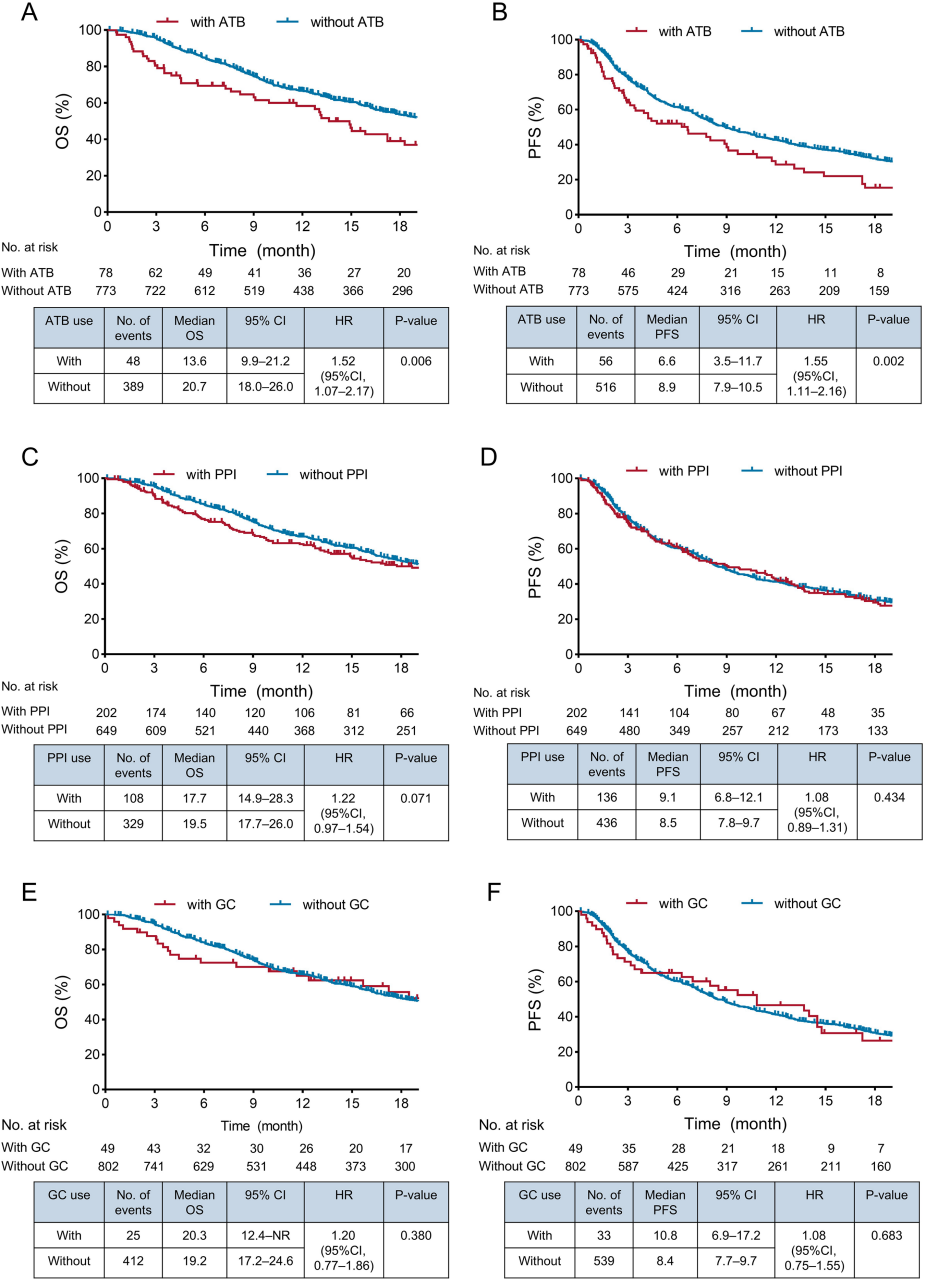
Survival outcomes of hepatocellular carcinoma patients with or without concomitant use of antibiotics, PPIs, or glucocorticoids. Kaplan-Meier curves showed that OS and PFS in patients with antibiotic use were significantly shorter than in patients without antibiotic use **(A, B)**. There was no difference in OS and PFS in patients with or without PPIs **(C, D)** and in those with glucocorticoids or not **(E, F)**. ATB, antibiotic; CI, confidence interval; GC, glucocorticoid; HR, hazard ratio; OS, overall survival; PFS, progression-free survival; PPI, proton pump inhibitor.

**Figure 2:**
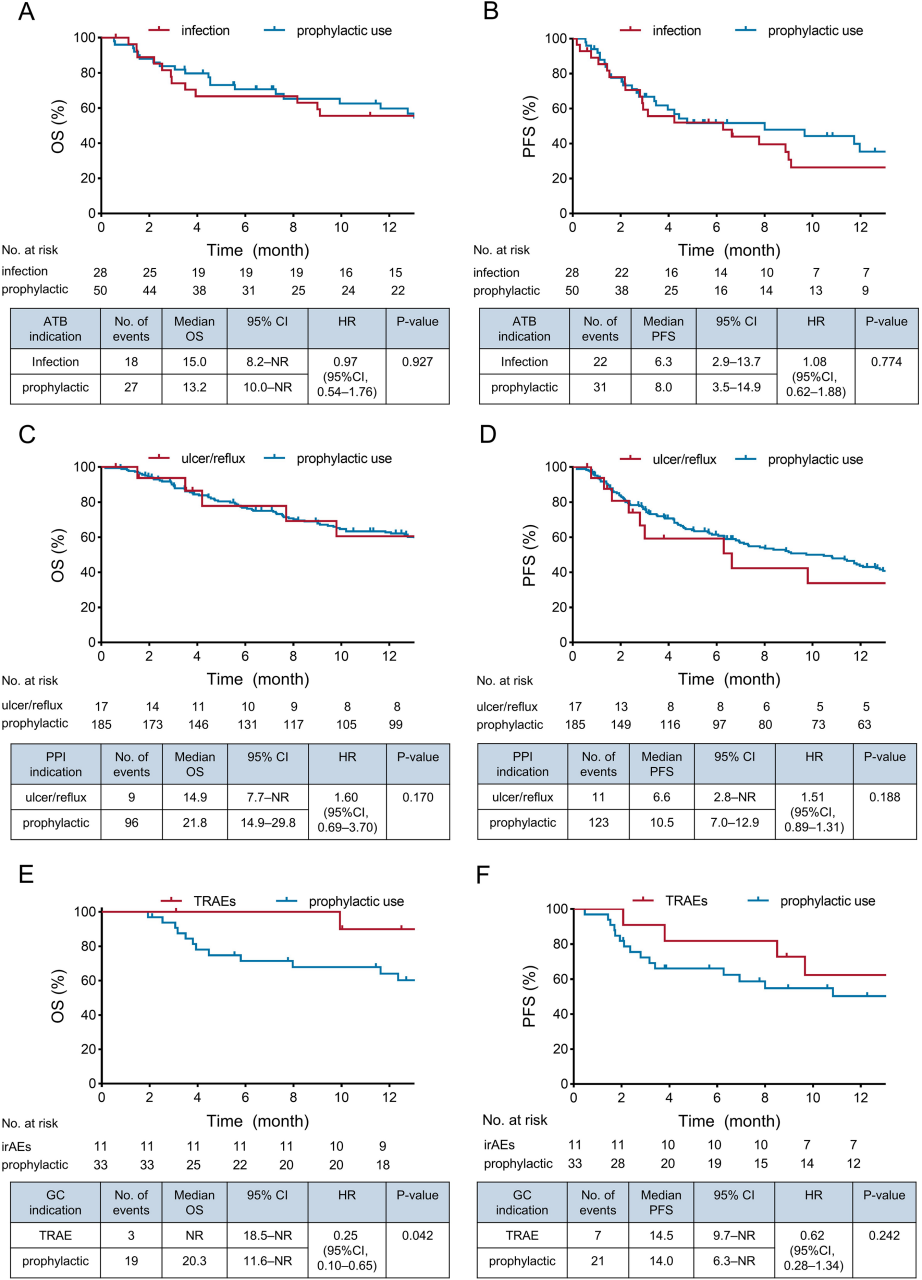
Survival outcomes of hepatocellular carcinoma patients receiving antibiotics, proton pump inhibitors, or glucocorticoids based on different indications. Kaplan-Meier curves showed that there was no difference in OS and PFS in patients receiving antibiotics for infection or prophylactic use **(A, B)** and in those receiving PPIs for ulcer/gastroesophageal reflux or prophylactic use **(C, D)**. Patients receiving glucocorticoids for early TRAE management had longer OS than those receiving prophylactic glucocorticoids **(E)**, while PFS did not differ between the two groups **(F)**. ATB, antibiotic; CI, confidence interval; GC, glucocorticoid; HR, hazard ratio; OS, overall survival; PFS, progression-free survival; PPI, proton pump inhibitor.

At the initiation of ICI treatment, antibiotics were prescribed to 78 patients (9.2%). Survival analyses showed a significant decrease in median OS, PFS, and TTP in antibiotic-exposed patients compared to non-antibiotic patients (OS: 13.6 vs. 20.7 months; P = 0.006; PFS: 6.6 vs. 8.9 months; P = 0.002; TTP: 8.0 vs 12.9 months, P = 0.012) (**Figures 1A, B**, **Supplementary Figure S1A**). No difference in tumor response, according to the best overall response (BOR), was observed between antibiotic-exposed patients and non-antibiotic patients (ORR: 15.4% vs. 17.7%; P = 0.605; DCR: 75.6% vs. 78.3%; P = 0.594). However, when tumor response was evaluated based on the first imaging follow-up, antibiotic-exposed patients obtained a significantly lower ORR (6.4% vs. 13.8%; P = 0.013) (**Supplementary Figure S2**). In terms of indications of antibiotic use, antibiotics were utilized for treating diagnosed infectious diseases or for prophylactic/empirical purposes. The latter involved preventing potential infections associated with invasive procedures (e.g., tumor biopsies), and managing suspected infectious symptoms without a formal diagnosis. Survival outcomes did not significantly differ between patients treated for anti-infection and those treated for prophylactic use (OS: 15.0 vs. 13.2 months; P = 0.927; PFS: 6.3 vs. 8.0 months; P = 0.774) (**Figures 2A, B**). Besides, similar OS and PFS were observed between patients with antibiotic exposure exceeding 2 weeks and those with exposure less than 2 weeks (OS: 13.2 vs. 15.0 months; P = 0.623; PFS: 4.2 vs. 7.8 months; P = 0.512) (**Supplementary Figure S3**). Among antibiotic-exposed patients, the type of antibiotics included cephalosporins (n = 44; 56.4%), penicillins (n = 14; 17.9%), and fluoroquinolones (n = 20; 25.6%) (**Supplementary Table S5**). Comparable survival was observed among groups receiving different types of antibiotics (OS: P = 0.587; PFS: P = 0.108) (**Supplementary Figure S3**). Additionally, 26.9% (21/78) of patients who received concomitant antibiotics required subsequent antibiotic treatment during ICI therapy, compared to 13.8% (107/773) of those without concomitant antibiotic exposure (**Supplementary Table S5**).

**Figure 3:**
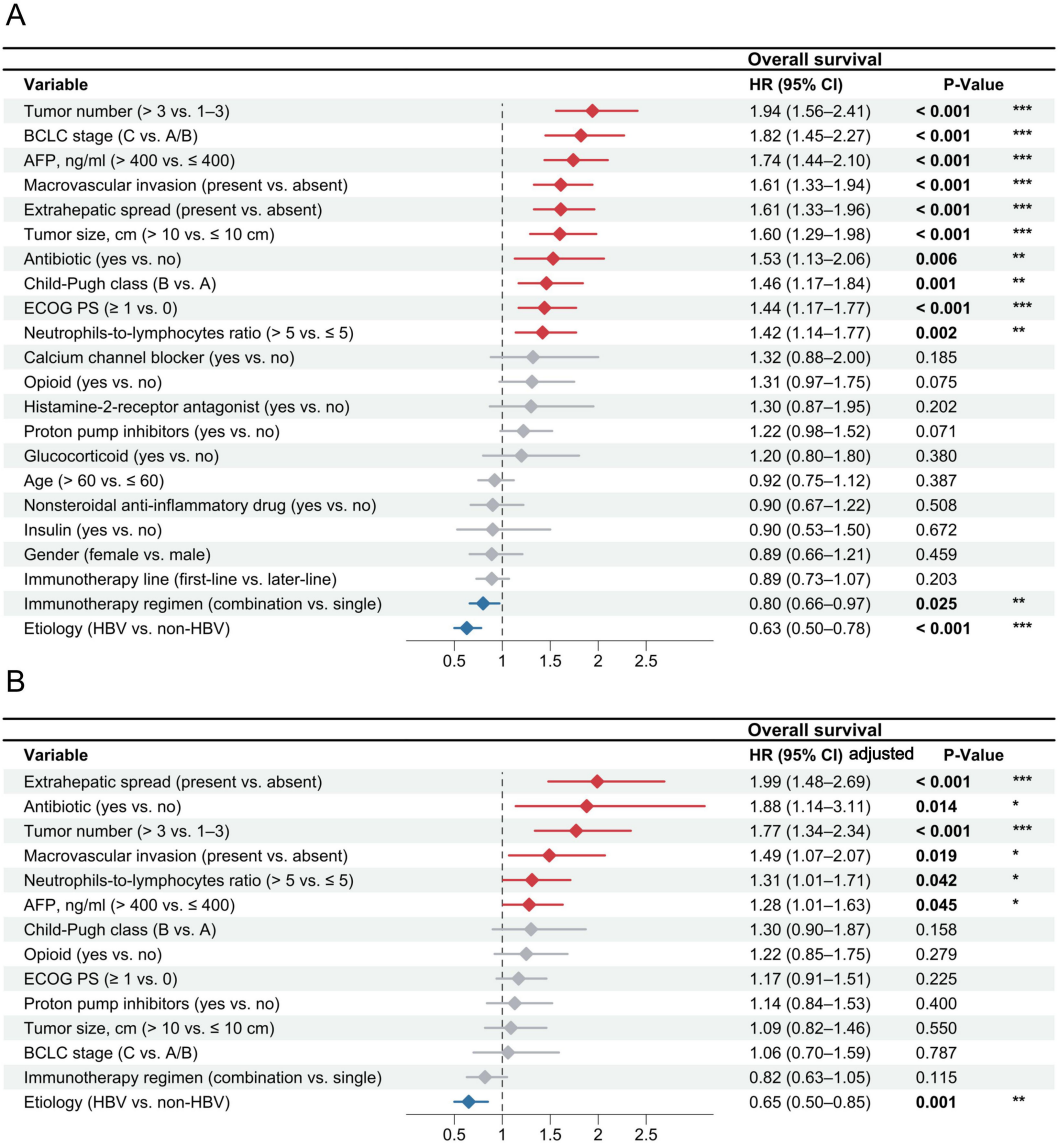
Univariable and multivariable Cox regression analyses for overall survival of hepatocellular carcinoma patients treated with immune-based therapy. The association between clinical variables and overall survival was evaluated through univariable Cox regression analyses **(A)** and multivariable Cox regression analyses **(B)**. AFP, α-fetoprotein; BCLC, Barcelona Clinic Liver Cancer; ECOG PS, Eastern Cooperative Oncology Group performance status; HBV, hepatitis B virus; HR, hazard ratio. * P < 0.05; ** P < 0.01; *** P < 0.001.

**Figure 4:**
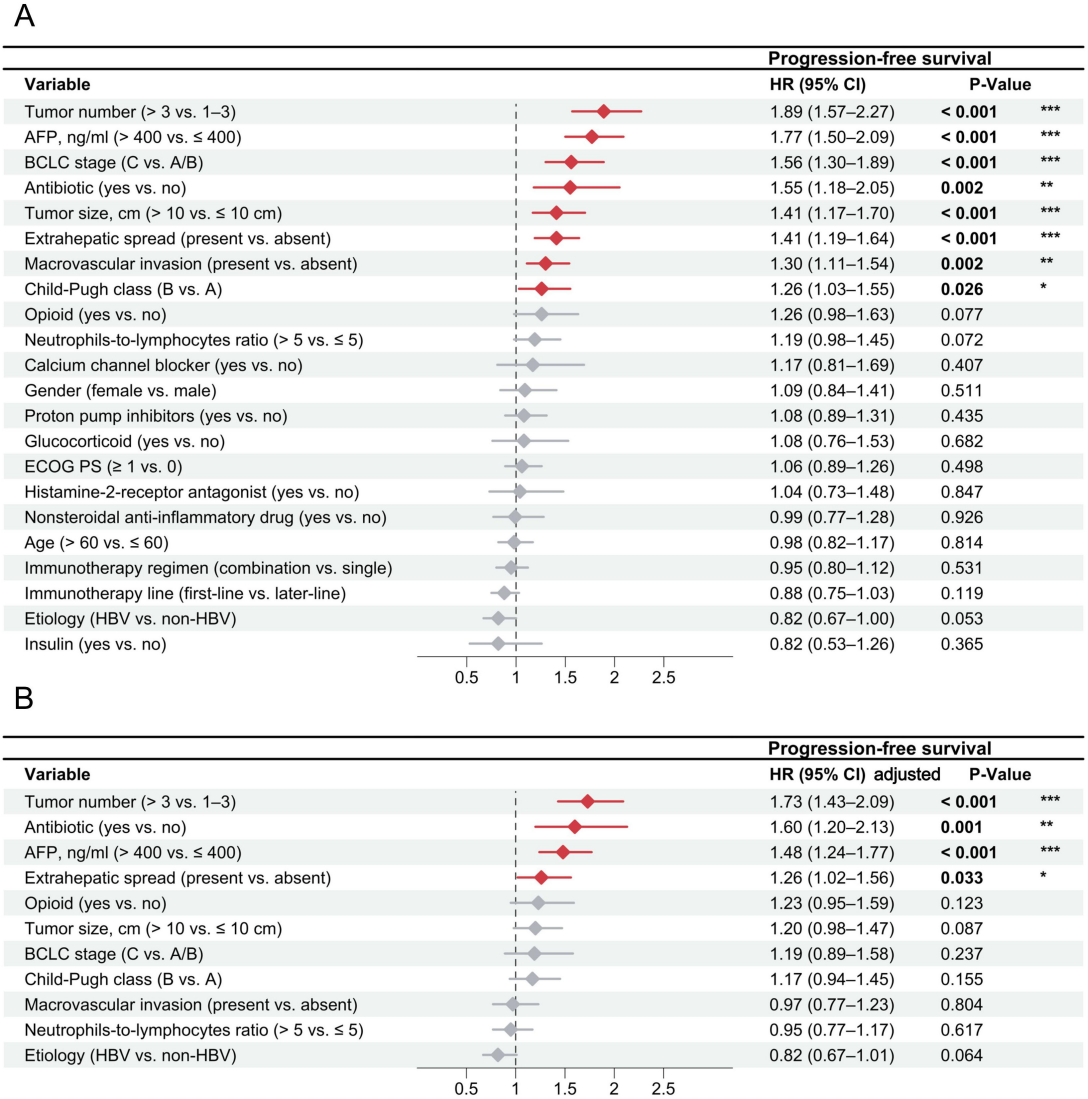
Univariable and multivariable Cox regression analyses for progression-free survival of HCC patients treated with immune-based therapy. The association between clinical variables and progression-free survival was evaluated through univariable Cox regression analyses **(A)** and multivariable Cox regression analyses **(B)**. AFP, α-fetoprotein; BCLC, Barcelona Clinic Liver Cancer; ECOG PS, Eastern Cooperative Oncology Group performance status; HBV, hepatitis B virus; HR, hazard ratio. * P < 0.05; ** P < 0.01; *** P < 0.001.

In the multivariable analysis of OS (**Figure 3**), patients with concomitant antibiotic use (HR = 1.88, 95% CI, 1.14–3.11; P = 0.014), ≥ 3 intrahepatic lesions (HR = 1.77, 95% CI, 1.34–2.34; P < 0.001), AFP > 400 ng/ml (HR = 1.28, 95%CI, 1.01–1.63; P = 0.045), macrovascular invasion (HR = 1.49, 95%CI, 1.07–2.07; P = 0.019), extrahepatic spread (HR = 1.99, 95% CI, 1.48–2.69; P < 0.001), and NLR > 5 (OS: HR = 1.31, 95%CI, 1.01–1.71; P = 0.042) were identified to have higher risks of death. Conversely, HBV etiology (HR = 0.65, 95% CI, 0.50–0.85; P < 0.001) was associated with a lower risk of death. For PFS, concomitant antibiotic use (HR = 1.60, 95% CI, 1.20–2.13; P = 0.001), ≥ 3 intrahepatic lesions (HR = 1.73, 95% CI, 1.43–2.09; P < 0.001), AFP > 400 ng/ml (HR = 1.48, 95%CI, 1.24–1.77; P < 0.001), and extrahepatic spread (HR = 1.27, 95% CI, 1.02–1.56; P = 0.033) were risk factors of disease progression (**Figure 4**). For TTP, concomitant antibiotic use (HR = 1.62, 95% CI, 1.17–2.23; P = 0.003), ≥ 3 intrahepatic lesions (HR = 1.63, 95% CI, 1.32–2.01; P < 0.001), AFP > 400 ng/ml (HR = 1.60, 95% CI, 1.31–1.96; P < 0.001), and extrahepatic spread (HR = 1.29, 95% CI, 1.04–1.60; P = 0.022) were also the independent risk factors (**Supplementary Figure S4**). After the 30-day landmark selection, multivariable analyses of survival outcomes further demonstrated the relationship between antibiotic exposure and decreased OS (HR = 1.54, 95% CI, 1.10–2.14; P = 0.012), PFS (HR = 1.58, 95% CI, 1.17–2.12; P = 0.003), and TTP (HR = 1.55, 95% CI, 1.11–2.18; P = 0.010) (**Supplementary Table S6**). We also calculated the E-values of concomitant antibiotic use for OS, PFS, and TTP, and compared them to the hazard ratios of established risk factors for these outcomes. This comparison suggests that it is unlikely an unmeasured confounder could fully account for the observed associations between antibiotic use and treatment outcomes in HCC patients treated with ICIs (**Supplementary Table S7**). Regarding tumor response based on BOR, logistic regression models showed no significant association between concomitant medication use and either the ORR or DCR (**Supplementary Tables S8**, **S9**). Notably, when evaluating tumor response at the first imaging follow-up, antibiotic use was independently associated with a lower ORR (HR = 0.61, 95% CI, 0.42–0.95; P = 0.041) (**Supplementary Tables S8**, **S9**).

To further validate the negative impacts of concomitant antibiotic use on survival outcomes, we conducted ancillary analyses in several restricted subgroups (**Table 2**). In the Child-Pugh class A subgroup, antibiotic use was significantly associated with reduced OS (P = 0.028), PFS (P = 0.027), and TTP (P = 0.045). Antibiotic use was significantly associated with reduced OS (P = 0.003), PFS (P = 0.030), and TTP (P = 0.023) in patients receiving ICI monotherapy, and with shorter PFS (P <0.001) and TTP (P = 0.001) in those treated with ICI-based combination therapy. The adverse effects of antibiotic use on PFS (P = 0.030) and TTP (P = 0.022) were also observed among patients treated with first-line ICIs (**Table 2**).

**Table 2:** The relationship between concomitant antibiotic use and efficacy outcomes of restricted subgroups of HCC patients.

	OS, months	HR (95%CI), P value	PFS, months	HR (95%CI), P value	TTP, months	HR (95%CI), P value
Analysis restricted to patients with Child-Pugh A liver function						
With antibiotic	13.6 (11.6–34.2)	1.52 (1.04–2.21); P = 0.028	8.0 (4.2–11.7)	1.47 (1.04–2.07); P = 0.027	8.9 (4.8–17.4)	1.48 (1.02–2.16); P = 0.045
Without antibiotic	22.8 (19.0–28.3)		9.7 (8.4–11.3)		13.3 (11.2–16.2)	
Analysis restricted to patients who underwent ICI monotherapy						
With antibiotic	9.9 (4.5–29.0)	1.77 (1.10–2.84); P = 0.003	6.6 (2.8–13.1)	1.52 (1.04–2.23); P = 0.030	6.7 (3.5–NR)	1.65 (1.07–2.56); P = 0.023
Without antibiotic	18.5 (15.7–24.6)		8.2 (6.9–10.8)		13.3 (9.5–20.60)	
Analysis restricted to patients who underwent ICI-based combination therapy						
With antibiotic	17.2 (13.0–NR)	1.51 (0.90–2.53); P = 0.122	6.3 (3.5–12.0)	2.10 (1.39–3.17); P <0.001	7.8 (4.2–NR)	2.15 (1.36–3.39); P = 0.001
Without antibiotic	23.0 (18.4–28.8)		9.5 (8.1–11.2)		14.0 (11.3–17.2)	
Analysis restricted to patients who underwent first-line ICI therapy						
With antibiotic	17.2 (12.8–NR)	1.50 (0.99–2.28); P = 0.055	8.9 (3.5–13.1)	1.50 (1.04–2.18); P = 0.030	8.9 (6.3–25.8)	1.64 (1.07–2.50); P = 0.022
Without antibiotic	23 (18.5–29.8)		9.3 (7.9–11.8)		14.8 (11.4–21.7)	

HR, hazard ratios; OS, overall survival; PFS, progression-free survival; TTP, time to progression.

### Proton pump inhibitor use shows no significant association with efficacy

The survival outcomes were comparable between patients treated (n = 202; 23.7%) or not treated with PPIs (OS: 17.7 vs. 19.5 months; P = 0.071; PFS: 9.1 vs. 8.5 months; P = 0.434; TTP: 12.8 vs. 12.6 months; P = 0.414) (**Figures 1C, D**, **Supplementary Figures S1B, C**), and tumor response did not differ between the two groups (BOR: ORR: 21.7% vs. 16.2%; P = 0.068; DCR: 82.2% vs. 76.7%; P = 0.104) (**Supplementary Figure S2**). PPIs were prescribed for both prophylactic and symptomatic purposes. Prophylactic use mainly included the prevention of gastric acid-related mucosal injury or bleeding. Symptomatic use primarily involved the management of gastrointestinal disorders such as peptic ulcer disease, gastroesophageal reflux, or immunotherapy-related gastrointestinal adverse events (e.g., abdominal pain, dyspepsia, or vomiting). Among patients treated with PPIs due to either ulcer/gastroesophageal reflux (n = 17; 8.4%) or prophylactic use (n = 185; 91.6%) (**Supplementary Table S10**), similar survival outcomes were noted between groups (**Figures 2C, D**). Both the PPI type (OS: P = 0.282; PFS: P = 0.321) and duration of PPI prescription (OS: P = 0.354; PFS: P = 0.941) did not impact the survival outcomes (**Supplementary Figure S5**).

### Glucocorticoids for TRAEs treatment is related to improved survival

Glucocorticoids were prescribed to 49 patients (5.8%), mainly for prophylactic use (n = 33; 67.3%) and for early management of TRAE occurring within 30 days of ICI initiation (n = 11; 22.4%). Comparing patients receiving glucocorticoids or not, the median OS, PFS, and TTP between two groups were 20.3 versus 19.2 months (P = 0.380), 10.8 versus 8.4 months (P = 0.683), and 14.0 versus 12.6 months (P = 0.758), respectively (**Figures 1E, F**, **Supplementary Figure S1D**), and the tumor response was also similar between groups (BOR: ORR: 18.4% vs. 17.5%; P = 0.871; DCR: 77.6% vs. 78.1%; P = 0.934) (**Supplementary Figure S2**). To assess whether glucocorticoid dosage or treatment duration had an impact on patient outcomes, we categorized patients into groups receiving > 50 mg/d versus < 50 mg/d and > 5 days versus < 5 days. Survival analyses showed no difference in OS and PFS based on daily dosage and duration (P > 0.05 for all) (**Supplementary Figure S6**).

Of note, the 11 patients using glucocorticoids for early TRAEs had a significantly longer median OS compared to those treated for prophylactic use (OS: Not reached vs. 20.3 months; P = 0.042) (**Figures 2E, F**). To overcome the immortal-time bias, further clinical outcomes analysis was performed after a 30-day landmark selection, including only patients with a minimum ICI treatment duration of 30 days (**Supplementary Figure S7**, 43 patients are included). Consistently, superior survival benefits were observed in patients using glucocorticoids for early TRAEs compared to those for prophylactic use (P = 0.048). Among the 11 patients, four patients had elevated liver enzyme levels (36.4%) and three had immune-related rash (27.3%). The majority of these patients (n = 8; 72.7%) were still alive (3 with rash, 2 with elevated liver enzyme level, 1 with pneumonia, 1 with hyperthyroidism, and 1 with both hypoalbuminemia and fever). The three patients experiencing rash achieved relatively long-term survival (Patient 1: 40.0 months; Patient 2: 27.7 months; Patient 3: 23.7 months) (**Supplementary Table S11**).

### Other concomitant medications show variable associations with efficacy

Other medications, including NSAIDs (n = 99; 11.6%), opioids (n = 98; 11.5%), H2RAs (n = 50; 5.8%), CCBs (n = 46; 5.4%), and insulins (n = 31; 3.6%), were prescribed in our cohort. Comparable survival and tumor response were observed between groups treated or not treated with CCBs and insulins. Patients with H2RAs had an increased ORR (based on BOR) than non-H2RAs patients (32.0% vs. 16.6%; P = 0.007), and patients with NSAIDs or opioids had a decreased DCR (based on first imaging follow-up) than those without (NSAIDs: 68.3% vs. 80.3%, P = 0.018; Opioids: 68.1% vs. 80.1%, P = 0.026), while no difference in survival was observed (**Supplementary Figures S1**, **2**, **8**, **9**).

### Polypharmacy shows no significant association with efficacy

Furthermore, we assessed the impacts of polypharmacy of different concomitant medications on survival outcomes. No difference in survival outcomes was found among subgroups with different numbers of concomitant medications (**Supplementary Figure S10**). In terms of specific combinations of different concomitant medications, 42 of 361 patients (11.6%) received both antibiotics and PPIs, 22 (6.1%) with PPIs and glucocorticoids, and 15 (4.2%) with antibiotics and glucocorticoids. All such combinations had no impact on survival when compared to single-agent use or no use (**Supplementary Figure S11**).

### Relationship between concomitant medications and immunotherapy safety

**Figure 5:**
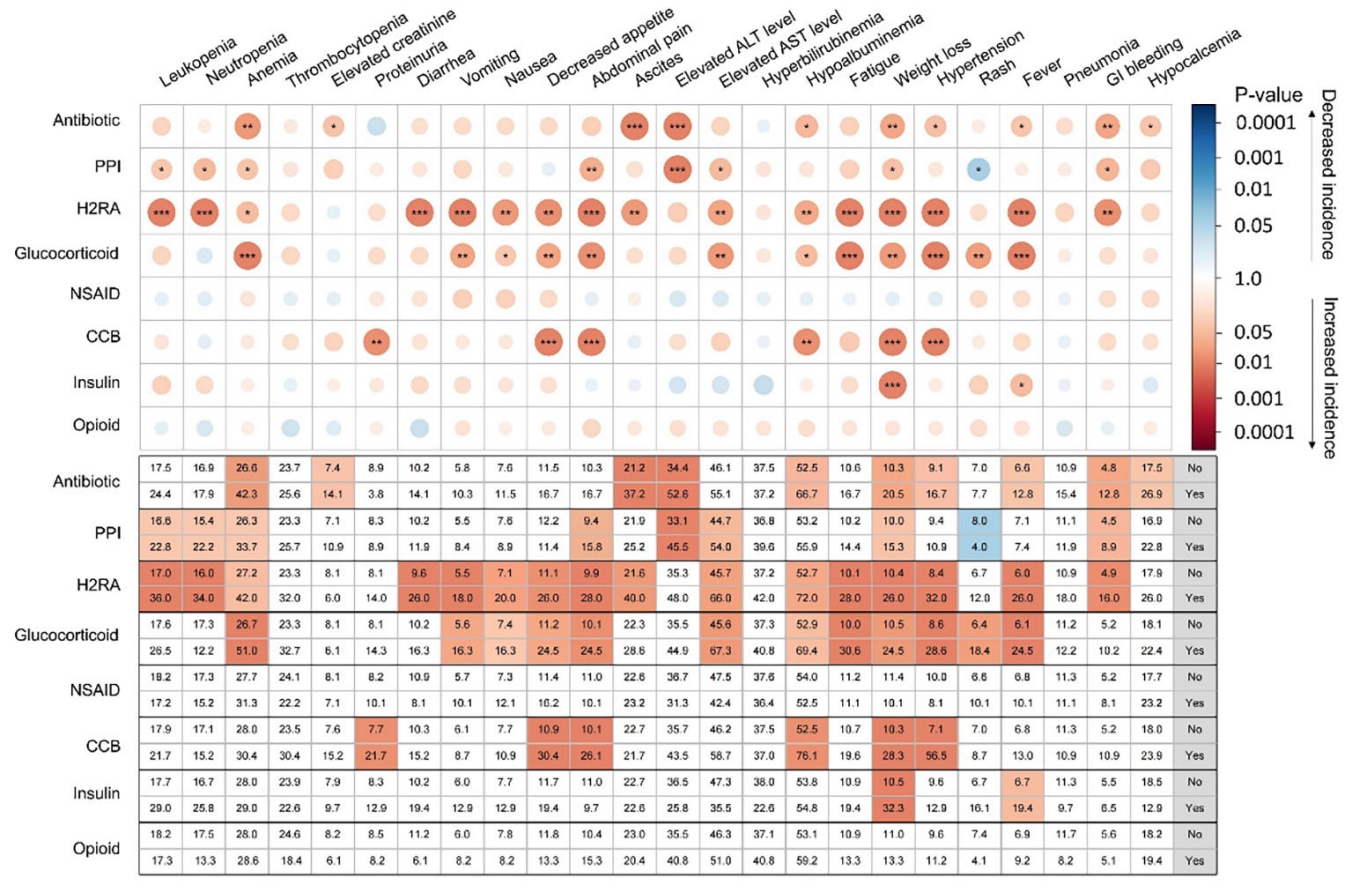
Summary of the associations between each drug category and incidence of various types of treatment-related adverse events using χ^2^-test. The size and color depth of the circle in the matrix correspond to the significance of statistical difference (P-value), and the table below shows the incidence data in detail. * P < 0.05; ** P < 0.01; *** P < 0.001.

Overall, 752 patients (88.4%) developed at least one TRAE during immunotherapy, and 248 patients (29.1%) experienced grade ≥ 3 TRAEs. Details of TRAEs are presented in **Supplementary Table S12**. In the multivariable analyses, Child-Pugh class was the only factor independently associated with a higher incidence of TRAEs of any grade (OR = 4.34, 95% CI, 1.82–10.00; P < 0.001) and grade ≥ 3 (OR = 1.49, 95% CI, 1.04–2.14; P = 0.032) (**Supplementary Table S13**). Concomitant use of H2RAs (OR = 1.83, 95% CI, 1.02–3.28; P = 0.042) and antibiotics (OR = 1.79, 95% CI, 1.02–3.28; P = 0.017) was positively associated with the development of grade ≥ 3 TRAEs in univariable analysis, but not in multivariable analysis. Additionally, use of antibiotics, PPIs, H2RA, glucocorticoids, CCBs, and insulins was related to increased incidence of certain TRAEs involving hematological, hepatic, and endocrine systems. The incidence of anemia, hypoalbuminemia, and hypertension was significantly higher in patients receiving antibiotics, H2RAs, or glucocorticoids (**Figure 5**).

## Discussion

**Figure 6:**
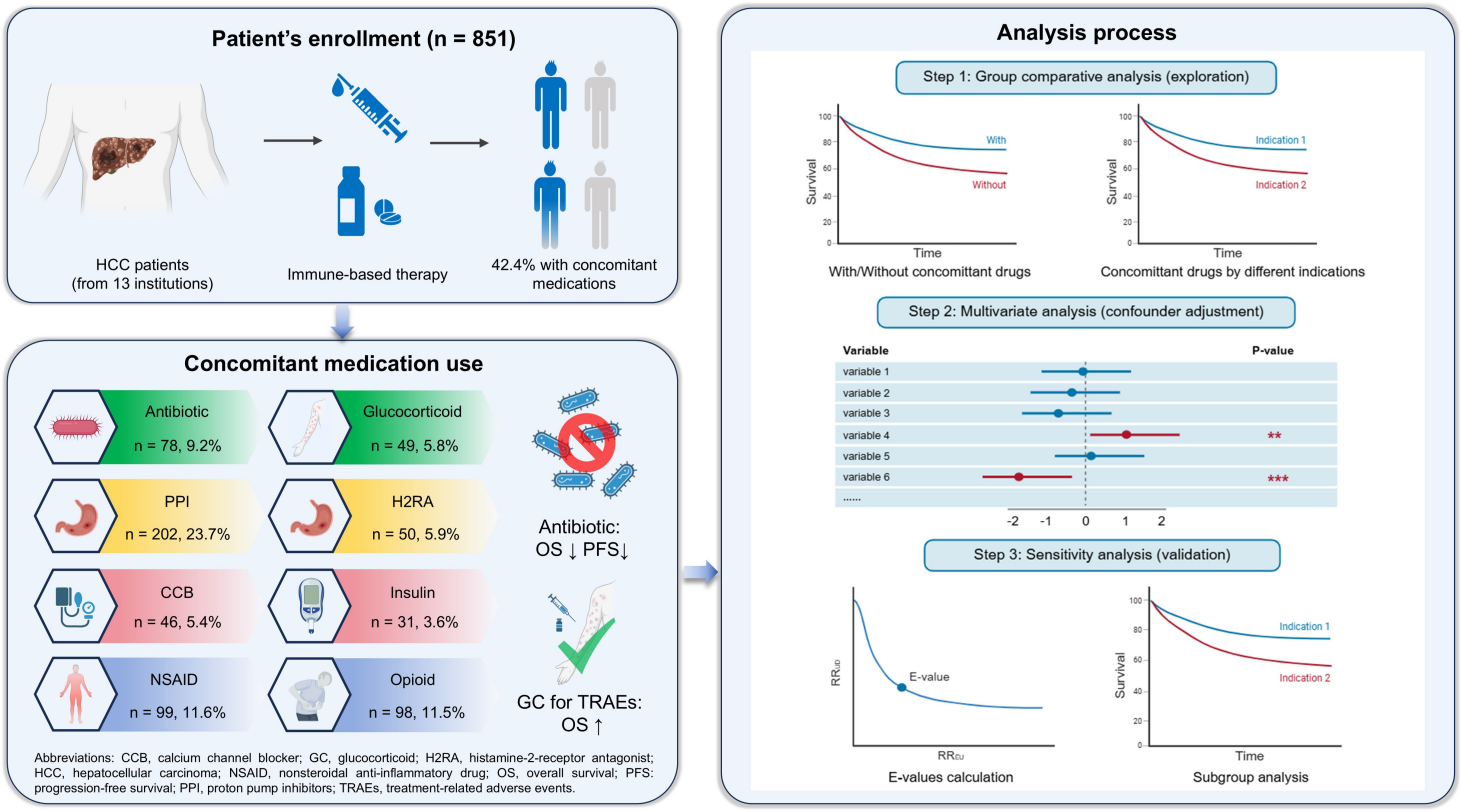
Summary of the study. The impact of concomitant medication use on survival outcomes in patients with hepatocellular carcinoma receiving immune-based therapy was evaluated using Kaplan-Meier curves and multivariate analysis, with results further validated through sensitivity analysis.

In this multicenter cohort study, we performed an integrated analysis of the association between concomitant use of multiple common medications and the clinical outcomes in a nationwide HCC population undergoing immune-based therapy. Our results highlighted the detrimental effects of antibiotics on survival outcomes in patients undergoing immunotherapy. Concomitant glucocorticoid use for early TRAE management may indicate improved survival benefits from ICIs. We also explored the impacts of other concomitant medications and observed no significant association with patient survival. Regarding safety profiles, multiple concomitant medications, such as antibiotics, H2RAs, and glucocorticoids, were associated with an increased occurrence of various TRAEs. Our findings emphasize the importance of carefully evaluating concomitant medications, particularly antibiotic prescriptions, when initiating immune-based therapy in HCC. To the best of our knowledge, this study represents the first comprehensive analysis of commonly prescribed medications and the clinical outcomes following immunotherapy for HCC patients (**Figure 6**).

In our study, antibiotic use at the initiation of ICI therapy was identified as a risk factor for OS and PFS after adjusting for other potential confounders. Analyses of tumor response based on BOR further showed that patients receiving concomitant antibiotics exhibited lower ORR and DCR compared to those without antibiotic exposure, although this discrepancy did not reach statistical significance. Notably, the first post-treatment imaging follow-up showed a significantly reduced ORR among the antibiotic group (**Supplementary Table S8**). These findings imply that antibiotic use may delay the onset of ICI efficacy, thereby potentially compromising the overall treatment outcomes. Consistent with our findings, a population-based study by Pinato et al. reported the negative association between early antibiotic exposure and outcomes among HCC patients receiving immunotherapy. However, the detrimental effect of antibiotic exposure differed between ICI monotherapy and combinations with TKI or anti-VEGF agents. Antibiotic use was significantly associated with reduced PFS and OS in the ICI monotherapy cohort, whereas in the combination cohort, the association was observed only for PFS and not for OS (32). Moreover, Hatanaka et al. analyzed a Japanese cohort treated with atezolizumab plus bevacizumab and found no significant difference in survival outcomes between antibiotic-exposed and non-exposed patients after adjusting for baseline characteristics. This regimen-related discrepancy may be partly attributed to the immunomodulatory effects of TKIs or anti-VEGF agents, as well as the significantly prolonged OS associated with combination therapy. Specifically, the addition of TKIs and anti-VEGF agents may enhance immune cell infiltration by normalizing tumor vasculature and reducing the accumulation of immunosuppressive cells (e.g., MDSCs and Tregs), thereby fostering a more immunosupportive tumor microenvironment. These findings suggest that the immune-modulating effects of TKIs and anti-VEGF agents may mitigate the adverse impact of antibiotics on ICI treatment in the HCC population. Furthermore, since ICI-based combination therapy significantly prolongs OS in HCC patients (37), this extended survival benefit may narrow the outcome gap between antibiotic-exposed and non-exposed patients, potentially underestimating the detrimental effect of antibiotic use.

The detrimental effects of concomitant antibiotic use on immunotherapy outcomes are thought to be associated with the perturbation of gut microbiota (17, 38–41). Preclinical evidence suggests that antibiotic use can reduce gut microbiota diversity and suppress their toll-like receptor signaling, leading to decreased pro-inflammatory cytokine production. This impairs the maturation of antigen-presenting cells and the activation of CD8+ T cells, ultimately affecting the initiation of anti-tumor immune response (39, 42). Accumulating evidence suggests that the timing of antibiotic exposure may play a critical role in modulating the efficacy of immune checkpoint inhibitors, likely through transient disruption of gut microbial diversity during the early phase of immune priming. Derosa et al. reported that antibiotic use within 30 days before ICI initiation was significantly associated with worse survival outcomes, while extending the exposure window to 60 days diminished this effect (34). These findings support the hypothesis that peri-treatment dysbiosis may interfere with immune activation and antitumor responses. It is conceivable that initiating ICIs after a period of microbiome recovery—particularly following empiric or prophylactic antibiotic use—might help mitigate the detrimental effects on immune efficacy. However, the appropriate timing for initiating or delaying ICIs in such settings remains undefined. Future prospective studies incorporating microbial profiling and immune monitoring are warranted to elucidate the optimal antibiotic-to-ICI interval and to inform evidence-based treatment sequencing in patients who require antibiotics.

Currently, antibiotic use for active infection at initiation of treatment has been designated as an exclusion criterion in numerous phase 3 clinical trials investigating ICI therapy for HCC, whereas the use of prophylactic antibiotics has not been emphasized (6, 43–45). In our study, a reduction of both OS and PFS was observed in patients who received antibiotics. Subgroup analyses based on the indications for antibiotic use revealed no significant difference in survival outcomes between patients treated for active infections and those using drugs for prophylaxis. Thus, beyond the management of active infection, caution should be exercised in the prophylactic use of antibiotics in both real-world clinical practice and future HCC immunotherapy trials.

In various malignancies, gastric acid suppressants have been implicated in impairing host anti-tumor immunity through inducing gut microbiota alterations (14, 30, 46, 47). However, in the context of HCC, previous studies have suggested that the efficacy of ICIs remains unaffected by the use of gastric acid suppressants (31, 48). A multi-center study involving 441 HCC patients receiving atezolizumab plus bevacizumab reported comparable PFS and OS between patients with and without PPI treatment (31). Similarly, another study found no compromised outcomes among HCC patients receiving ICIs in conjunction with concomitant PPI or H2RA therapy (48). In our study, we conducted a comprehensive assessment of PPIs, considering treatment indications, drug types, and duration, and found no evidence that concomitant PPI use attenuates the efficacy of immunotherapy. Moreover, in cirrhotic HCC patients, particularly those with portal hypertension, prophylactic PPIs may mitigate the risk of ulcer perforation and variceal bleeding, thereby facilitating the long-term use of ICI.

Several studies have reported that cancer patients receiving ICIs and concomitant glucocorticoids exhibit similar survival outcomes compared to glucocorticoid non-users (14, 29). Cortellini et al. reported that corticosteroid use within 30 days prior to ICI initiation and during ICI treatment did not influence the response and survival of HCC patients receiving PD-1/PD-L1 inhibitors (49). Consistent with these findings, our study also found comparable survival outcomes between patients with and without concomitant corticosteroid exposure. Nevertheless, subgroup analysis based on glucocorticoids indications revealed that patients receiving glucocorticoids for early TRAE management, such as rash and elevated liver enzyme level, exhibited longer OS and PFS than those receiving prophylactic glucocorticoids. A previous study described a correlation between the development of grade ≥ 2 TRAEs, such as dermatologic and endocrine reactions, and improved outcomes in HCC patients undergoing ICI therapy (50). Theoretically, preclinical research has identified multiple epitopes shared by tumor cells and parenchymal tissues, suggesting a mechanism whereby shared antigens of tumor cells released by ICI therapy could prime a secondary immune response against host normal tissues (51). Therefore, the occurrence of early TRAEs reflects a robust immune reaction against both tumor cells and normal tissues, supporting the notion that early TRAEs may serve as a prognostic factor in HCC patients treated with immunotherapy (52).

At present, the potential impact of concomitant medications, such as opioids, NSAIDs, and insulins, on the ICI efficacy in cancer patients remains uncertain (53–55). A retrospective study reported no association between survival outcomes and the use of opioids and antidiabetics (53) while another study suggested that concomitant use of opioids and antidiabetics was associated with shortened OS in cancer patients receiving ICIs (55). In the context of HCC, we investigated the relationship between these concomitant medications and immunotherapy outcomes and found that these medications did not impact either survival or tumor response in multivariable models.

In addition, our study observed a higher incidence of various types of TRAEs, such as elevated liver enzyme levels, among patients receiving concomitant medications. It is important to emphasize that these findings indicate only an association between concomitant medication use and the occurrence of specific TRAEs, without establishing a direct causal relationship between medication use and an increase in TRAE incidence. Notably, concomitant medications, including opioids, NSAIDs, antibiotics, and gastric acid suppressants, have been reported to cause drug accumulation and increase systemic toxicity by impairing liver function (56). Therefore, such concomitant medications should be used with caution in HCC patients receiving immunotherapy, especially those with decompensated liver function.

Several limitations were presented in our study. Firstly, studies evaluating the impact of concomitant medications are inherently susceptible to clinical heterogeneity and residual confounding. Although we applied multivariable Cox models and performed sensitivity analyses to mitigate these effects, a cautious interpretation of our findings is still warranted. Secondly, the observational nature of this study precluded us from determining appropriate sample sizes and pre-defined stratification based on the concomitant medications. Furthermore, the absence of biochemical validation experiments limited the mechanistic evidence supporting the impact of concomitant medication use on the immune microenvironment and immunotherapy in HCC. Thirdly, consistent with other retrospective studies, patients with incomplete imaging or laboratory data (either at baseline or during post-treatment examination) and those lost to follow-up were excluded. These patients were more likely to have advanced disease, poorer performance status, or suboptimal treatment adherence, which may have introduced selection bias and limited the generalizability of our findings. Therefore, our results should be interpreted with caution, and future prospective studies with more comprehensive data collection are warranted to further validate these findings. Fourthly, as HBV infection was the predominant etiology in our cohort, the generalizability of our findings to non-HBV-related HCC populations (e.g., HCV-, metabolic-, or alcohol-associated HCC) may be limited. Nevertheless, similar associations between antibiotic use and reduced ICI efficacy have been reported in studies primarily involving non-HBV populations (32), suggesting that these relationships may extend beyond a single etiologic background. Further large-scale studies focusing on non-HBV-related HCC are warranted to more comprehensively elucidate the impact of concomitant medications on immunotherapy outcomes. Fourthly, the subgroup analyses based on specific clinical indications for concomitant medication use were exploratory in nature and conducted on relatively small sample sizes, which limits the statistical power for definitive conclusions. Therefore, these findings should be interpreted with appropriate caution.

## Conclusions

In conclusion, our study underscored the negative impact of antibiotics on the prognosis of HCC patients treated with ICIs. While a potential association between early glucocorticoid use for TRAE management and improved survival was observed, this finding was based on a limited sample size and warrants further validation. Concomitant medications may elevate the risk of various types of TRAEs, emphasizing the importance of careful prescription practices when administering these drugs to HCC patients receiving immune-based therapies.

## Data Availability

The datasets presented in this study can be found in online repositories. The names of the repository/repositories and accession number(s) can be found below: The clinical dataset is available upon request as per the controlled access approach of the Research Data Deposit website platform of Sun Yat-sen University Cancer Center (RDDA2025415407).
