# Supplementary material for: Concomitant medication use and clinical outcome in hepatocellular carcinoma treated with immune-based therapy: a multicenter analysis
Source: Front Immunol. 2025 Nov 28;16:1680015. doi: 10.3389/fimmu.2025.1680015 (PMC12698624; doi:10.3389/fimmu.2025.1680015)
Supplement: Supplementary file 1 [file Table1.docx]

**Supplementary appendix**

Supplement to: **Concomitant medication use and clinical outcome in hepatocellular carcinoma treated with immune-based therapy: a multicenter retrospective study**

**Table of content**

[Table S1. Study institutes. 2](#_Toc10709)

[Table S2. Details of the immunotherapy regimen. 3](#_Toc28920)

[Table S3. Duration of medication and time of medication administration relative to ICI initiation. 4](#_Toc17289)

[Table S4. Summary of associations between each drug category and patient characteristics. 5](#_Toc29398)

[Table S5. Additional details on the concomitant antibiotics. 11](#_Toc21282)

[Table S6. Multivariable Cox regression analyses for efficacy outcomes of hepatocellular carcinoma patients treated with immune-based therapy after 30-day landmark selection. 12](#_Toc32277)

[Table S7. E-value for the association between antibiotics use and survival outcomes in adjusted Cox models. 13](#_Toc5735)

[Table S8.Univariable and multivariable logistic regression analysis for overall response rate of HCC patients treated with immune-based therapy. 15](#_Toc19426)

[Table S9. Univariable and multivariable logistic regression analysis for disease control rate of HCC patients treated with immune-based therapy. 18](#_Toc4680)

[Table S10. Additional details on the concomitant gastric acid suppressants. 21](#_Toc9216)

[Table S11. Summary of survival data and occurred TRAEs of patients who underwent concomitant glucocorticoid use for TRAE management. 22](#_Toc13262)

[Table S12. Summary of TRAEs among HCC patients who underwent immune-based therapy. 24](#_Toc6773)

[Table S13. Univariable and multivariable analyses illustrating the relationship between baseline medication exposure and treatment-related toxicity in HCC patients with HCC treated with immune-based therapy. 26](#_Toc13688)

[Figure S1. Time to progression of hepatocellular carcinoma patients with or without concomitant use of commonly prescribed medications. 28](#_Toc1015)

[Figure S2. Overall tumor response regarding concomitant medication according to the Response Evaluation Criteria In Solid Tumors (RECIST) 1.1 criteria. 29](#_Toc21652)

[Figure S3. Subgroup analyses on survival outcomes in hepatocellular carcinoma patients with concomitant antibiotic use. 30](#_Toc7772)

[Figure S4. Univariable and multivariable Cox regression analyses for time to progression of hepatocellular carcinoma patients treated with immune-based therapy. 31](#_Toc32346)

[Figure S5. Subgroup analyses on survival outcomes in hepatocellular carcinoma patients with concomitant proton pump inhibitor use. 32](#_Toc5585)

[Figure S6. Subgroup analyses on survival outcomes in hepatocellular carcinoma patients with concomitant glucocorticoid use. 33](#_Toc6858)

[Figure S7. 30-day landmark analyses of survival outcomes of hepatocellular carcinoma patients receiving glucocorticoids based on different indications. 34](#_Toc5300)

[Figure S8. Overall survival of hepatocellular carcinoma patients with or without concomitant use of other commonly prescribed medications. 35](#_Toc2979)

[Figure S9. Progression-free survival of hepatocellular carcinoma patients with or without concomitant use of other commonly prescribed medications. 36](#_Toc15164)

[Figure S10. Subgroup analyses on survival outcomes in patients based on the different number of concomitant medications. 37](#_Toc26606)

[Figure S11. Subgroup analyses on survival outcomes in hepatocellular carcinoma patients based on different concomitant medication combinations. 38](#_Toc1362)

# Table S1. Study institutes.

| Institute | Actual Number  (N = 851) |
| --- | --- |
| Boai Hospital of Zhongshan | 12 |
| Chengdu Seventh People's Hospital | 15 |
| Chenzhou Municipal Hospital of Traditional Chinese Medicine | 26 |
| Ganzhou Cancer Hospital | 20 |
| Guangyuan Central Hospital | 13 |
| Guangzhou Chest Hospital | 19 |
| Jiangmen Hospital of Traditional Chinese Medicine | 26 |
| Sun Yat-sen University Cancer Center | 566 |
| The First Affiliated Hospital of Hainan Medical University | 95 |
| The First Hospital of Nanchang | 17 |
| Wuchuan People's Hospital | 15 |
| Yantai Hospital of Traditional Chinese Medicine | 12 |
| Yunan County People's Hospital | 15 |

# Table S2. Details of the immunotherapy regimen.

| Treatment regimen | No.(%) |
| --- | --- |
| Anti-PD-1/PD-L1 monotherapy | 316 (37.1) |
| Pembrolizumab | 64 (7.5) |
| Tislelizumab | 252 (29.6) |
| Anti-PD-1/PD-L1 + TKIs | 315 (37.0) |
| Pembrolizumab + lenvatinib | 48 (5.6) |
| Tislelizumab + lenvatinib | 256 (30.1) |
| Durvalumab + lenvatinib | 11 (1.3) |
| Anti-PD-1/PD-L1 + anti-angiogenic agents | 220 (25.9) |
| Atezolizumab + bevacizumab | 76 (8.9) |
| Sintilimab + bevacizumab | 144 (16.9) |

# Table S3. Duration of medication and time of medication administration relative to ICI initiation.

| Concomitant medications | duration of medication  median, IQR (week) | time of medication administration relative to ICI initiation median, IQR (week) |
| --- | --- | --- |
| Antibiotic | 1.42 (1.20–8.24) | 0.00 (-0.30–0.13) |
| Proton pump inhibitor | 2.26 (1.20–19.95) | 0.00 (0.00–1.59) |
| Histamine-2-receptor antagonist | 4.50 (1.20–25.00) | 0.00 (0.00–3.13) |
| Glucocorticoid | 0.86 (0.43–14.16) | 1.59 (0.00–2.70) |
| NSAID | 1.37 (1.20–7.64) | 0.99 (0.13–2.88) |
| Calcium channel blocker | 0.30 (0.30–8.28) | 2.7 (0.13–3.00) |
| Opioid | 1.37 (0.30–4.20) | 0.00 (0.00–1.16) |

Abbreviations: ICI, immune checkpoint inhibitors; IQR, interquartile range; NSAIDs, nonsteroidal anti-inflammatory drug; PPI, proton pump inhibitors.

# Table S4. Summary of associations between each drug category and patient characteristics.

|  | ATB | | PPI | | H2RA | | GC | | NSAID | | CCB | | Insulin | | Opioid | |
| --- | --- | --- | --- | --- | --- | --- | --- | --- | --- | --- | --- | --- | --- | --- | --- | --- |
|  | No | Yes | No | Yes | No | Yes | No | Yes | No | Yes | No | Yes | No | Yes | No | Yes |
| Age, n (%) |  |  |  |  |  |  |  |  |  |  |  |  |  |  |  |  |
| > 60 | 254  (32.9) | 35  (44.9) | 224 (34.5) | 65 (32.2) | 274  (34.2) | 15  (30.0) | 275  (34.3) | 14  (28.6) | 260  (34.6) | 29  (29.3) | 270  (33.5) | 19  (41.3) | 279  (34.0) | 10  (32.3) | 257  (34.1) | 32  (32.7) |
| ≤ 60 | 519  (67.1) | 43  (55.1) | 425  (65.5) | 137 (67.8) | 527  (65.8) | 35  (70.0) | 527  (65.7) | 35  (71.4) | 492  (65.4) | 70  (70.7) | 535  (66.5) | 27  (58.7) | 541  (66.0) | 21  (67.7) | 496  (65.9) | 66  (67.3) |
| χ^2^ (P-value) | 0.033 | | 0.540 | | 0.542 | | 0.412 | | 0.297 | | 0.279 | | 0.838 | | 0.771 | |
| Gender, n (%) |  |  |  |  |  |  |  |  |  |  |  |  |  |  |  |  |
| Male | 686  (88.7) | 67  (85.9) | 576 (88.8) | 177 (87.6) | 707  (88.3) | 46  (92.0) | 714  (89.0) | 39  (79.6) | 665  (88.4) | 88  (88.9) | 715  (88.8) | 38  (82.6) | 725  (88.4) | 28  (90.3) | 666  (88.4) | 87  (88.8) |
| Female | 87  (11.3) | 11  (14.1) | 73 (11.2) | 25 (12.4) | 94  (11.7) | 4  (8.0) | 88  (11.0) | 10  (20.4) | 87  (11.6) | 11  (11.1) | 90  (11.2) | 8  (17.4) | 95  (11.6) | 3  (9.7) | 87  (11.6) | 11  (11.2) |
| χ^2^ (P-value) | 0.453 | | 0.661 | | 0.422 | | 0.045 | | 0.893 | | 0.199 | | 0.968 | | 0.923 | |
| Etiology, n (%) |  |  |  |  |  |  |  |  |  |  |  |  |  |  |  |  |
| HBV | 636  (82.3) | 56  (71.8) | 529 (81.5) | 163 (80.7) | 646  (80.6) | 46  (92.0) | 650  (81.0) | 42  (85.7) | 606  (80.6) | 86  (86.9) | 650  (80.7) | 42  (91.3) | 664  (81.0) | 28  (90.3) | 605  (80.3) | 87  (88.8) |
| Other | 137  (17.7) | 22  (28.2) | 120 (18.5) | 39 (19.3) | 155  (19.4) | 4  (8.0) | 152  (19.0) | 7  (14.3) | 146  (19.4) | 13  (13.1) | 155  (19.3) | 4  (8.7) | 156  (19.0) | 3  (9.7) | 148  (19.7) | 11  (11.2) |
| χ^2^ (P-value) | 0.024 | | 0.795 | | 0.046 | | 0.416 | | 0.132 | | 0.074 | | 0.190 | | 0.044 | |
| Tumor size, n (%) |  |  |  |  |  |  |  |  |  |  |  |  |  |  |  |  |
| ≤ 10 cm | 596  (77.1) | 56  (71.8) | 503 (77.5) | 149 (73.8) | 620  (77.4) | 32  (64.0) | 615  (76.7) | 37  (75.5) | 585  (77.8) | 67  (67.7) | 619  (76.9) | 33  (71.7) | 626  (76.3) | 26  (83.9) | 581  (77.2) | 71  (72.4) |
| > 10 cm | 177  (22.9) | 22  (28.2) | 146 (22.5) | 53 (26.2) | 181  (22.6) | 18  (36.0) | 187  (23.3) | 12  (24.5) | 167  (22.2) | 32  (32.3) | 186  (23.1) | 13  (28.3) | 194  (23.7) | 5  (16.1) | 172  (22.8) | 27  (27.6) |
| χ^2^ (P-value) | 0.291 | | 0.273 | | 0.030 | | 0.851 | | 0.025 | | 0.422 | | 0.331 | | 0.300 | |
| Tumor number, n (%) |  |  |  |  |  |  |  |  |  |  |  |  |  |  |  |  |
| 1–3 | 258  (33.4) | 31  (39.7) | 218 (33.6) | 71 (35.1) | 266  (33.2) | 23  (46.0) | 265  (33.0) | 24  (49.0) | 255  (33.9) | 34  (34.3) | 269  (33.4) | 20  (43.5) | 275  (33.5) | 14  (45.2) | 252  (33.5) | 37  (37.8) |
| > 3 | 515  (66.6) | 47  (60.3) | 431 (66.4) | 131 (64.9) | 535  (66.8) | 27  (54.0) | 537  (67.0) | 25  (51.0) | 497  (66.1) | 65  (65.7) | 536  (66.6) | 26  (56.5) | 545  (66.5) | 17  (54.8) | 501  (66.5) | 61  (62.2) |
| χ^2^ (P-value) | 0.258 | | 0.683 | | 0.064 | | 0.022 | | 0.932 | | 0.161 | | 0.180 | | 0.399 | |
| AFP, n (%) |  |  |  |  |  |  |  |  |  |  |  |  |  |  |  |  |
| < 400 ng/mL | 410  (53.0) | 40  (51.3) | 347  (53.5) | 103  (51.0) | 427  (53.3) | 23  (46.0) | 424  (52.9) | 26  (53.1) | 403  (53.6) | 47  (47.5) | 434  (53.9) | 16  (34.8) | 437  (53.3) | 13  (41.9) | 411  (54.6) | 39  (39.8) |
| ≥ 400 ng/mL | 363  (47.0) | 38  (48.7) | 302  (46.5) | 99  (49.0) | 374  (46.7) | 27  (54.0) | 378  (47.1) | 23  (46.9) | 349  (46.4) | 52  (52.5) | 371  (46.1) | 30  (65.2) | 383  (46.7) | 18  (58.1) | 342  (45.4) | 59  (60.2) |
| χ^2^ (P-value) | 0.767 | | 0.538 | | 0.315 | | 0.979 | | 0.252 | | 0.011 | | 0.214 | | 0.006 | |
| MVI, n (%) |  |  |  |  |  |  |  |  |  |  |  |  |  |  |  |  |
| Absence | 433  (56.0) | 48  (61.5) | 361 (55.6) | 120 (59.4) | 448  (55.9) | 33  (66.0) | 451  (56.2) | 30  (61.2) | 433  (57.6) | 48  (48.5) | 452  (56.1) | 29  (63.0) | 463  (56.5) | 18  (58.1) | 430  (57.1) | 51  (52.0) |
| Presence | 340  (44.0) | 30  (38.5) | 288 (44.4) | 82 (40.6) | 353  (44.1) | 17  (34.0) | 351  (43.8) | 19  (38.8) | 319  (42.4) | 51  (51.5) | 353  (43.9) | 17  (37.0) | 357  (43.5) | 13  (41.9) | 323  (42.9) | 47  (48.0) |
| χ^2^ (P-value) | 0.348 | | 0.344 | | 0.163 | | 0.494 | | 0.086 | | 0.359 | | 0.860 | | 0.341 | |
| EHS, n (%) |  |  |  |  |  |  |  |  |  |  |  |  |  |  |  |  |
| Absence | 448  (58.0) | 55  (70.5) | 365 (56.2) | 138 (68.3) | 470  (58.7) | 33  (66.0) | 475  (59.2) | 28  (57.1) | 437  (58.1) | 66  (66.7) | 470  (58.4) | 33  (71.7) | 481  (58.7) | 22  (71.0) | 446  (59.2) | 57  (58.2) |
| Presence | 325  (42.0) | 23  (29.5) | 284 (43.8) | 64 (31.7) | 331  (41.3) | 17  (34.0) | 327  (40.8) | 21  (42.9) | 315  (41.9) | 33  (33.3) | 335  (41.6) | 13  (28.3) | 339  (41.3) | 9  (29.0) | 307  (40.8) | 41  (41.8) |
| χ^2^ (P-value) | 0.032 | | 0.002 | | 0.307 | | 0.773 | | 0.104 | | 0.073 | | 0.171 | | 0.840 | |
| ECOG PS, n (%) |  |  |  |  |  |  |  |  |  |  |  |  |  |  |  |  |
| 0 | 265  (34.3) | 21  (26.9) | 219  (33.7) | 67  (33.2) | 275  (34.3) | 11  (22.0) | 271  (33.8) | 15  (30.6) | 255  (33.9) | 31  (31.3) | 277  (34.4) | 9  (19.6) | 276  (33.7) | 10  (32.3) | 249  (33.1) | 37  (37.8) |
| ≥ 1 | 508  (65.7) | 57  (73.1) | 430  (66.3) | 135  (66.8) | 526  (65.7) | 39  (78.0) | 531  (66.2) | 34  (69.4) | 497  (66.1) | 68  (68.7) | 528  (65.6) | 37  (80.4) | 544  (66.3) | 21  (67.7) | 504  (66.9) | 61  (62.2) |
| χ^2^ (P-value) | 0.190 | | 0.880 | | 0.073 | | 0.648 | | 0.607 | | 0.038 | | 0.871 | | 0.355 | |
| Prior surgery or local therapy, n (%) |  |  |  |  |  |  |  |  |  |  |  |  |  |  |  |  |
| Absence | 459 (59.3) | 45 (57.6) | 385 (59.3) | 119 (58.9) | 468 (58.4) | 36 (72.0) | 476 (59.3) | 28 (57.1) | 438 (58.2) | 66 (66.6) | 475 (59.0) | 29 (63.0) | 484 (59.0) | 20 (64.5) | 458 (60.8) | 46 (46.9) |
| Presence | 314 (40.6) | 33 (42.3) | 264 (40.6) | 83 (41.0) | 333 (41.5) | 14 (28.0) | 326 (40.6) | 21 (42.8) | 314 (41.7) | 33 (33.3) | 330 (40.9) | 17 (36.9) | 336 (40.9) | 11 (35.4) | 295 (39.1) | 52 (53.0) |
| χ^2^ (P-value) | 0.773 |  | 0.917 |  | 0.061 |  | 0.760 |  | 0.110 |  | 0.588 |  | 0.542 |  | 0.009 |  |
| Immunotherapy type, n (%) |  |  |  |  |  |  |  |  |  |  |  |  |  |  |  |  |
| Single | 272  (35.2) | 44  (56.4) | 218 (33.6) | 98 (48.5) | 297  (37.1) | 19  (38.0) | 298  (37.2) | 18  (36.7) | 283  (37.6) | 31  (31.3) | 303  (37.6) | 13  (28.3) | 307  (37.4) | 9  (29.0) | 278  (36.9) | 38  (38.8) |
| Combination | 501  (64.8) | 34  (43.6) | 431 (66.4) | 104 (51.5) | 504  (62.9) | 31  (62.0) | 504  (62.8) | 31  (63.3) | 469  (62.4) | 68  (68.7) | 502  (62.4) | 33  (71.7) | 513  (62.6) | 22  (71.0) | 475  (63.1) | 60  (61.2) |
| χ^2^ (P-value) | <0.001 | | < 0.001 | | 0.896 | | 0.953 | | 0.405 | | 0.200 | | 0.342 | | 0.720 | |
| Immunotherapy line, n (%) |  |  |  |  |  |  |  |  |  |  |  |  |  |  |  |  |
| First line | 425  (55.0) | 43  (55.1) | 356 (54.9) | 112 (55.4) | 433  (54.1) | 35  (70.0) | 440  (54.9) | 28  (57.1) | 404  (53.7) | 64  (64.6) | 439  (54.5) | 29  (63.0) | 450  (54.9) | 18  (58.1) | 422  (56.0) | 46  (46.9) |
| Second or subsequent line | 348  (45.0) | 35  (44.9) | 293 (45.1) | 90 (44.6) | 368  (45.9) | 15  (30.0) | 362  (45.1) | 21  (42.9) | 348  (46.3) | 35  (35.4) | 366  (45.5) | 17  (37.0) | 370  (45.1) | 13  (41.9) | 331  (44.0) | 52  (53.1) |
| χ^2^ (P-value) | 0.980 | | 0.883 | | 0.028 | | 0.755 | | 0.040 | | 0.259 | | 0.726 | | 0.088 | |

Abbreviations: ATB, antibiotic; BCLC, Barcelona Clinic Liver Cancer; CCB, calcium channel blocker; ECOG PS, Eastern Cooperative Oncology Group performance status; EHS, extrahepatic spread; GC, glucocorticoid; H2RA, histamine-2-receptor antagonist; MVI, Macrovascular invasion; NSAIDs, nonsteroidal anti-inflammatory drug; PPI, proton pump inhibitors.

# Table S5. Additional details on the concomitant antibiotics.

|  | N (%) |
| --- | --- |
| Type |  |
| Cephalosporins | 44 (56.4) |
| Penicillins | 14 (17.9) |
| Fluoroquinolones | 20 (25.6) |
| Indication |  |
| Empirical/prophylactic use | 50 (64.1) |
| Infection | 28 (35.9) |
| Pneumonia | 9 (11.5) |
| Gastro-intestinal infections | 5 (6.4) |
| Urinary tract infections | 4 (5.1) |
| Skin infections | 2 (2.6) |
| Other | 8 (10.3) |
| Patients treated with subsequent antibiotic during ICI therapy |  |
| Group with concomitant antibiotics (n = 78) | 21 (26.9) |
| Group without concomitant antibiotics (n = 773) | 107 (13.8) |

#

| **Variable** | **Overall Survival** | **Progression-free Survival** | **Time to progression** |
| --- | --- | --- | --- |
|  | Multivariable*  HR (95% CI); P-value | Multivariable*  HR (95% CI); P-value | Multivariable*  HR (95% CI); P-value |
| Antibiotic (yes vs. no) | 1.54 (1.10–2.14); P = 0.012 | 1.58 (1.17–2.12); P = 0.003 | 1.55 (1.11–2.18); P = 0.010 |
| Etiology (HBV vs. non-HBV) | 0.60 (0.48–0.76); P < 0.001 | 0.83 (0.67–1.02); P = 0.082 | -- |
| ECOG PS (≥ 1 vs. 0) | 1.16 (0.92–1.46); P = 0.200 | -- | -- |
| Child-Pugh class (B vs. A) | 1.20 (0.94–1.54); P = 0.150 | -- | -- |
| BCLC stage (C vs. A/B) | 1.04 (0.73–1.46); P = 0.846 | 1.19 (0.89–1.59); P = 0.246 | 1.07 (0.83–1.37); P = 0.622 |
| AFP, ng/ml (> 400 vs. ≤ 400) | 1.38 (1.12–1.70); P = 0.003 | 1.49 (1.24–1.78); P < 0.001 | 1.60 (1.31–1.97); P < 0.001 |
| Tumor diameter, cm (> 10 vs. ≤ 10) | 1.22 (0.96–1.56); P = 0.105 | 1.16 (0.94–1.43); P = 0.165 | 1.03 (0.81–1.31); P = 0.812 |
| Tumor number (> 3 vs. 1–3) | 1.68 (1.34–2.12); P < 0.001 | 1.74 (1.43–2.11); P < 0.001 | 1.63 (1.31–2.02);  P < 0.001 |
| Macrovascular invasion (present vs. absent) | 1.37 (1.03–1.82); P = 0.031 | 1.01 (0.79–1.29); P = 0.952 | -- |
| Extrahepatic spread (present vs. absent) | 1.68 (1.30–2.15); P < 0.001 | 1.23 (0.99–1.53); P = 0.064 | 1.27 (1.02–1.59); P = 0.034 |
| Treatment type (combination vs. single) | 0.79 (0.65–0.97); P = 0.021 | -- | -- |
| Treatment line (first-line vs. later-line) | -- | -- | -- |

# Table S6. Multivariable Cox regression analyses for efficacy outcomes of hepatocellular carcinoma patients treated with immune-based therapy after 30-day landmark selection.

*Only variable with P < 0.1 were included in the multivariate analyses.

# Table S7. E-value for the association between antibiotics use and survival outcomes in adjusted Cox models.

| Outcome | Adjusted HR (95% CI) for antibiotics use | E-value for HR estimate | Variable | Level | HR |
| --- | --- | --- | --- | --- | --- |
| OS | 1.88  (1.14–3.11) | 2.46 | Extrahepatic spread | present vs. absent | 1.99 |
|  |  |  | Tumor number | > 3 vs. 1–3 | 1.77 |
|  |  |  | Macrovascular invasion | present vs. absent | 1.49 |
|  |  |  | Neutrophils-to-lymphocytes ratio | > 5 vs. ≤ 5 | 1.31 |
|  |  |  | AFP, ng/ml | > 400 vs. ≤ 400 | 1.28 |
|  |  |  | Child-Pugh class | B vs. A | 1.30 |
|  |  |  | Opioid | yes vs. no | 1.25 |
|  |  |  | ECOG PS | ≥ 1 vs. 0 | 1.17 |
|  |  |  | Proton pump inhibitors | yes vs. no | 1.13 |
|  |  |  | Tumor size, cm | > 10 vs. ≤ 10 cm | 1.09 |
|  |  |  | BCLC stage | C vs. A/B | 1.06 |
|  |  |  | Immunotherapy regimen | combination vs. single | 0.82 |
|  |  |  | Etiology | HBV vs. non-HBV | 0.65 |
| PFS | 1.60  (1.20–2.13) | 2.11 | Tumor number | > 3 vs. 1–3 | 1.73 |
|  |  |  | Antibiotic | yes vs. no | 1.60 |
|  |  |  | AFP, ng/ml | > 400 vs. ≤ 400 | 1.48 |
|  |  |  | Extrahepatic spread | present vs. absent | 1.26 |
|  |  |  | Opioid | yes vs. no | 1.23 |
|  |  |  | Tumor size, cm | > 10 vs. ≤ 10 cm | 1.20 |
|  |  |  | BCLC stage | C vs. A/B | 1.19 |
|  |  |  | Child-Pugh class | B vs. A | 1.17 |
|  |  |  | Macrovascular invasion | present vs. absent | 0.97 |
|  |  |  | Neutrophils-to-lymphocytes ratio | > 5 vs. ≤ 5 | 0.95 |
| TTP | 1.62  (1.17–2.23) | 2.14 | Tumor number | > 3 vs. 1–3 | 1.63 |
|  |  |  | Antibiotic | yes vs. no | 1.62 |
|  |  |  | AFP, ng/ml | > 400 vs. ≤ 400 | 1.60 |
|  |  |  | Extrahepatic spread | present vs. absent | 1.29 |
|  |  |  | BCLC stage | C vs. A/B | 1.06 |

AFP, a-fetoprotein; BCLC, Barcelona Clinic Liver Cancer; ECOG PS, Eastern Cooperative Oncology Group performance status; HBV, hepatitis B virus; HR, hazard ratio; OS, overall survival; PFS, progression-free survival; TTP, time to progression.

# Table S8.Univariable and multivariable logistic regression analysis for overall response rate of HCC patients treated with immune-based therapy.

|  | **Objective response rate (best of response)** | | | | **Objective response rate (first follow-up imaging)** | | | |
| --- | --- | --- | --- | --- | --- | --- | --- | --- |
|  | **Univariate Analysis** | | **Multivariate Analysis** | | **Univariate Analysis** | | **Multivariate Analysis** | |
|  | **OR (95% CI)** | **P-value** | **OR (95% CI)** | **P-value** | **OR (95% CI)** | **P-value** | **OR (95% CI)** | **P-value** |
| Age (> 60 vs. ≤ 60) | 0.91 (0.62–1.33) | 0.620 |  |  | 0.82 (0.47–1.43) | 0.474 |  |  |
| Gender (female vs. Male) | 1.07 (0.62–1.84) | 0.812 |  |  | 1.27 (0.58–2.81) | 0.554 |  |  |
| Etiology (HBV vs. non-HBV) | 0.94 (0.60–1.47) | 0.788 |  |  | 2.00 (0.93–4.31) | 0.076 | 1.87 (0.79–3.34) | 0.224 |
| ECOG PS (≥ 1 vs. 0) | 0.93 (0.64–1.35) | 0.713 |  |  | 1.25 (0.73–2.15) | 0.412 |  |  |
| Child-Pugh class (B vs. A) | 0.76 (0.47–1.20) | 0.237 |  |  | 1.02 (0.47–2.24) | 0.958 |  |  |
| BCLC stage (C vs. A/B C) | 0.63 (0.43–0.90) | 0.012 | 0.88 (0.56–1.37) | 0.557 | 0.77 (0.46–1.34) | 0.360 |  |  |
| AFP, ng/ml (> 400 vs. ≤ 400) | 0.93 (0.65–1.33) | 0.690 |  |  | 0.95 (0.57–1.58) | 0.833 |  |  |
| Tumor size, cm (> 10 vs. ≤ 10) | 1.48 (1.00–2.19) | 0.052 | 1.54 (1.00–2.36) | 0.048 | 1.03 (0.57–1.86) | 0.936 |  |  |
| Tumor number (> 3 vs. 1–3) | 0.48 (0.33–0.69) | < 0.001 | 0.48 (0.33–0.69) | < 0.001 | 0.42 (0.36–0.65) | < 0.001 | 0.49 (0.38–0.67) | < 0.001 |
| Macrovascular invasion (Present vs. Absent) | 0.97 (0.68–1.39) | 0.887 |  |  | 0.70 (0.42–1.18) | 0.183 |  |  |
| Extrahepatic spread (Present vs. Absent) | 0.53 (0.36–0.78) | 0.001 | 0.61 (0.39–0.95) | 0.031 | 0.76 (0.48–1.06) | 0.088 | 0.81 (0.53–1.27) | 0.271 |
| Immunotherapy regimen (combination vs. single) | 1.35 (0.92–1.97) | 0.121 |  |  | 1.62 (1.06–2.63) | 0.017 | 1.71 (1.13–2.45) | 0.035 |
| Immunotherapy line (first-line vs. later-line) | 2.23 (1.52–3.27) | < 0.001 | 1.99 (1.34–2.94) | < 0.001 | 1.07 (0.64–1.79) | 0.794 |  |  |
| Antibiotic (yes vs. no) | 0.84 (0.44–1.60) | 0.605 |  |  | 0.52 (0.33–0.76) | 0.013 | 0.61 (0.42–0.95) | 0.041 |
| Proton pump inhibitor (yes vs. no) | 1.44 (0.97–2.14) | 0.068 | 1.26 (0.83–1.91) | 0.281 | 0.84 (0.44–1.63) | 0.605 |  |  |
| Histamine-2-receptor antagonist (yes vs. no) | 2.36 (1.27–4.41) | 0.007 | 1.71 (0.88–3.31) | 0.113 | 1.78 (0.38–8.41) | 0.468 |  |  |
| Glucocorticoid (yes vs. no) | 1.06 (0.51–2.24) | 0.871 |  |  | 0.49 (0.06–3.72) | 0.107 |  |  |
| Nonsteroidal anti-inflammatory drug (yes vs. no) | 0.97 (0.56–1.70) | 0.925 |  |  | 0.92 (0.42–2.01) | 0.833 |  |  |
| Calcium channel blocker (yes vs. no) | 1.16 (0.55–2.45) | 0.706 |  |  | 0.67 (0.87–1.35) | 0.193 |  |  |
| Insulin (yes vs. no) | 1.68 (0.73–3.82) | 0.220 |  |  | 1.72 (0.48–6.16) | 0.402 |  |  |
| Opioid (yes vs. no) | 1.24 (0.73–2.10) | 0.423 |  |  | 1.48 (0.72–3.07) | 0.289 |  |  |

AFP, a-fetoprotein; BCLC, Barcelona Clinic Liver Cancer; ECOG PS, Eastern Cooperative Oncology Group performance status; HBV, hepatitis B virus; OR, odds ratio.

# Table S9. Univariable and multivariable logistic regression analysis for disease control rate of HCC patients treated with immune-based therapy.

|  | **Disease control rate (best of response)** | | | | **Disease control rate (first follow-up imaging)** | | | |
| --- | --- | --- | --- | --- | --- | --- | --- | --- |
|  | **Univariate Analysis** | | **Multivariate Analysis** | | **Univariate Analysis** | | **Multivariate Analysis** | |
|  | **OR (95% CI)** | **P-value** | **OR (95% CI)** | **P-value** | **OR (95% CI)** | **P-value** | **OR (95% CI)** | **P-value** |
| Age (> 60 vs. ≤ 60) | 1.08 (0.77–1.52) | 0.661 |  |  | 1.37 (0.88–2.12) |  |  |  |
| Gender (female vs. Male) | 1.04 (0.62–1.72) | 0.890 |  |  | 0.84 (0.42–1.67) | 0.621 |  |  |
| Etiology (HBV vs. non-HBV) | 1.00 (0.66–1.52) | 0.990 |  |  | 1.54 (0.97–2.44) | 0.066 | 1.54 (0.96–2.50) | 0.076 |
| ECOG PS (≥ 1 vs. 0) | 1.20 (0.85–1.69) | 0.281 |  |  | 1.04 (0.78–1.45) | 0.245 |  |  |
| Child-Pugh class (B vs. A) | 0.45 (0.31–0.65) | < 0.001 | 0.47 (0.31–0.68) | < 0.001 | 0.66 (0.34–1.31) | 0.239 |  |  |
| BCLC stage (C vs. A/B C) | 0.46 (0.31–0.68) | < 0.001 | 0.88 (0.48–1.59) | 0.663 | 0.83 (0.52–1.31) | 0.413 |  |  |
| AFP, ng/ml (> 400 vs. ≤ 400) | 0.47 (0.34–0.66) | < 0.001 | 0.56 (0.40–0.81) | 0.002 | 0.66 (0.45–0.99) | 0.042 | 0.73 (0.48–1.10) | 0.132 |
| Tumor size, cm (> 10 vs. ≤ 10) | 0.85 (0.58–1.23) | 0.404 |  |  | 0.67 (0.41–1.11) | 0.120 |  |  |
| Tumor number (> 3 vs. 1–3) | 0.42 (0.28–0.62) | < 0.001 | 0.47 (0.31–0.71) | < 0.001 | 0.60 (0.38–0.95) | 0.043 | 0.61 (0.38–0.98) | 0.043 |
| Macrovascular invasion (Present vs. Absent) | 0.63 (0.45–0.87) | 0.005 | 0.93 (0.60–1.47) | 0.764 | 0.90 (0.6–1.33) | 0.592 |  |  |
| Extrahepatic spread (Present vs. Absent) | 0.50 (0.36–0.70) | < 0.001 | 0.55 (0.36–0.84) | 0.005 | 0.74 (0.50–1.10) | 0.131 |  |  |
| Immunotherapy regimen (combination vs. single) | 1.02 (0.72–1.43) | 0.923 |  |  | 1.75 (1.15–2.67) | 0.009 | 1.75 (1.14–2.70) | 0.011 |
| Immunotherapy line (first-line vs. later-line) | 1.05 (0.76–1.45) | 0.760 |  |  | 1.25 (0.84–1.85) | 0.270 |  |  |
| Antibiotic (yes vs. no) | 0.86 (0.50–1.49) | 0.594 |  |  | 0.83 (0.35–2.00) | 0.686 |  |  |
| Proton pump inhibitor (yes vs. no) | 1.39 (0.93–2.08) | 0.104 |  |  | 1.38 (0.87–2.20) | 0.178 |  |  |
| Histamine-2-receptor antagonist (yes vs. no) | 1.14 (0.56–2.33) | 0.728 |  |  | 1.21 (0.26–5.68) | 0.809 |  |  |
| Glucocorticoid (yes vs. no) | 1.03 (0.49–1.92) | 0.934 |  |  | 0.87 (0.28–2.71) | 0.809 |  |  |
| Nonsteroidal anti-inflammatory drug (yes vs. no) | 1.12 (0.67–1.89) | 0.651 |  |  | 0.53 (0.31–0.90) | 0.018 | 0.60 (0.34–1.06) | 0.079 |
| Calcium channel blocker (yes vs. no) | 1.16 (0.55–2.44) | 0.685 |  |  | 1.49 (0.33–6.80) | 0.610 |  |  |
| Insulin (yes vs. no) | 4.17 (1.00–16.67) | 0.050 | 4.00 (0.93–16.67) | 0.061 | 0.87 (037–3.60) | 0.870 |  |  |
| Opioid (yes vs. no) | 0.97 (0.62–1.61) | 0.904 |  |  | 0.53 (0.30–0.93) | 0.026 | 0.70 (0.38–1.31) | 0.270 |

AFP, a-fetoprotein; BCLC, Barcelona Clinic Liver Cancer; ECOG PS, Eastern Cooperative Oncology Group performance status; HBV, hepatitis B virus; OR, odds ratio.

# Table S10. Additional details on the concomitant gastric acid suppressants.

|  | N (%) |
| --- | --- |
| **Proton pump inhibitor** |  |
| Type |  |
| Ilaprazole | 44 (22.3) |
| Omeprazole | 75 (37.1) |
| Rabeprazole | 26 (12.9) |
| Lansoprazole | 38 (18.8) |
| Pantoprazole | 19 (9.4) |
| Indication |  |
| Prophylactic use | 185 (91.6) |
| Ulcer/Gastroesophageal reflux | 17 (8.4) |
| **Histamine-2-receptor antagonist** |  |
| Type |  |
| Cimetidine | 15 (30.0) |
| Ranitidine | 7 (14.0) |
| Famotidine | 28 (56.0) |
| Indication |  |
| Prophylactic use | 35 (70.0) |
| Ulcer/Gastroesophageal reflux | 15 (30.0) |

# Table S11. Summary of survival data and occurred TRAEs of patients who underwent concomitant glucocorticoid use for TRAE management.

| No. of patients | TRAE occurrence | OS (months) | PFS (months) | Tumor response | Status |
| --- | --- | --- | --- | --- | --- |
| 1 | Elevated ALT level (grade 4)  Elevated AST level (grade 4) | 10.0 | 8.9 | PR | Dead |
| 2 | Elevated ALT level (grade 4)  Elevated AST level (grade 4) | 10.0 | 9.7 | PR | Alive |
| 3 | Elevated ALT level (grade 2)  Elevated AST level (grade 2) | 18.5 | 8.5 | SD | Dead |
| 4 | Elevated AST level (grade 2) | 14.5 | 14.5 | SD | Alive |
| 5 | Rash (grade 3) | 27.7 | 14.5 | SD | Alive |
| 6 | Rash (grade 3) | 23.7 | 13.7 | PR | Alive |
| 7 | Rash (grade 2) | 40.0 | 34.0 | PR | Alive |
| 8 | Pneumonia (grade 2) | 33.8 | 32.2 | PR | Alive |
| 9 | Elevated creatinine (grade 2)  Proteinuria (grade 2) | 12.5 | 3.8 | PD | Dead |
| 10 | Hypoalbuminemia (grade 3)  Fever (grade 3) | 3.1 | 2.1 | PD | Alive |
| 11 | Hyperthyroidism (grade 2) | 14.9 | 14.9 | SD | Alive |

Abbreviations: ALT, alanine aminotransferase; AST, aspartate transaminase; OS, overall survival; PD, progression disease; PFS, progression-free survival; PR, partial response; SD, stable disease; TRAE, treatment-related adverse events.

# Table S12. Summary of TRAEs among HCC patients who underwent immune-based therapy.

| TRAEs | All treated patients (n = 851) | |
| --- | --- | --- |
|  | All grades, n (%) | Grade 3-4, n (%) |
| Any | 752 (88.4) | 248 (29.1) |
| Leukopenia | 154 (18.1) | 14 (1.6) |
| Neutropenia | 145 (17.0) | 13 (1.5) |
| Anemia | 239 (28.1) | 26 (3.1) |
| Thrombocytopenia | 203 (23.9) | 31 (3.6) |
| Elevated creatinine | 68 (8.0) | 2 (0.2) |
| Proteinuria | 72 (8.5) | 11 (1.3) |
| Diarrhea | 90 (10.6) | 11 (1.3) |
| Vomiting | 53 (6.2) | 0 |
| Nausea | 67 (7.9) | 0 |
| Decreased appetite | 102 (12.0) | 0 |
| Abdominal pain | 93 (10.9) | 10 (1.2) |
| Ascites | 193 (22.7) | 17 (2.0) |
| Elevated ALT level | 307 (36.1) | 35 (4.1) |
| Elevated AST level | 399 (46.9) | 55 (6.5) |
| Hyperbilirubinemia | 319 (37.5) | 43 (5.1) |
| Hypoalbuminemia | 458 (53.8) | 32 (3.8) |
| Fatigue | 95 (11.2) | 3 (0.4) |
| Weight loss | 96 (11.3) | 9 (1.1) |
| Hypertension | 83 (9.8) | 27 (3.2) |
| Rash | 60 (7.1) | 20 (2.4) |
| Fever | 51 (6.0) | 8 (0.9) |
| Pneumonia | 96 (11.3) | 12 (1.4) |
| Gastrointestinal bleeding | 47 (5.5) | 15 (1.8) |
| Hypocalcemia | 156 (18.3) | 1 (0.1) |
| Hyperthyroidism | 22 (2.6) | 0 |
| Hypothyroidism | 20 (2.3) | 0 |
| Dyspnea | 16 (1.9) | 4 (0.5) |
| Cardiac failure | 3 (0.4) | 2 (0.2) |
| Myocardial infarction | 2 (0.2) | 2 (0.2) |

Abbreviations: ALT, alanine aminotransferase; AST, aspartate transaminase; TRAE, treatment-related adverse events.

# Table S13. Univariable and multivariable analyses illustrating the relationship between baseline medication exposure and treatment-related toxicity in HCC patients with HCC treated with immune-based therapy.

|  | Any treatment-related adverse event | | | | Grade 3–4 treatment-related adverse event | | | |
| --- | --- | --- | --- | --- | --- | --- | --- | --- |
|  | Univariable Analysis | | Multivariable Analysis | | Univariable Analysis | | Multivariable Analysis | |
|  | OR (95% CI) | P | OR (95% CI) | P | OR (95% CI) | P | OR (95% CI) | P |
| Age (> 60 vs. ≤ 60) | 0.81 (0.52–1.23) | 0.323 |  |  | 0.97 (0.71–1.33) | 0.846 |  |  |
| Gender (female vs. male) | 1.04 (0.54–2.04) | 0.893 |  |  | 1.27 (0.81–1.99) | 0.295 |  |  |
| Etiology (HBV vs. non-HBV) | 1.20 (0.72–2.0) | 0.493 |  |  | 1.18 (0.80–1.75) | 0.402 |  |  |
| BCLC stage (C vs. A/B) | 1.79 (1.15–2.70) | 0.009 | 1.37 (0.66–2.86) | 0.40 | 1.33 (0.96–1.85) | 0.083 | 1.30 (0.86–1.96) | 0.210 |
| Child-Pugh class (B vs. A) | 4.35 (1.89–10.00) | < 0.001 | 4.34 (1.82–10.00) | < 0.001 | 1.63 (1.15–2.31) | 0.007 | 1.49 (1.04–2.14) | 0.032 |
| Tumor size, cm (> 10 vs. ≤ 10 cm) | 1.67 (0.95–2.94) | 0.073 | 1.47 (0.83–2.63) | 0.19 | 1.30 (0.91–1.85) | 0.155 |  |  |
| Tumor number (> 3 vs. 1–3) | 1.18 (0.77–1.82) | 0.446 |  |  | 0.93 (0.68–1.27) | 0.658 |  |  |
| AFP, ng/ml (> 400 vs. ≤ 400) | 1.12 (0.74–1.72) | 0.570 |  |  | 1.15 (0.86–1.55) | 0.354 |  |  |
| Macrovascular invasion (present vs. absent) | 1.47 (0.95–2.27) | 0.084 | 1.02 (0.53–1.96) | 0.96 | 1.29 (0.96–1.74) | 0.089 | 1.10 (0.76–1.60) | 0.601 |
| Extrahepatic spread (present vs absent) | 1.59 (1.02–2.50) | 0.040 | 1.36 (0.75–2.50) | 0.30 | 1.06 (0.79–1.43) | 0.692 |  |  |
| ECOG PS (≥ 1 vs. 0) | 1.38 (2.12–0.91) | 0.129 |  |  | 1.15 (0.84–1.58) | 0.393 |  |  |
| Immunotherapy regimen (combination vs single) | 1.22 (0.80–1.89) | 0.349 |  |  | 1.13 (0.83–1.54) | 0.427 |  |  |
| Immunotherapy line (first-line vs. later-line) | 0.81 (0.53–1.23) | 0.328 |  |  | 0.88 (0.66–1.19) | 0.414 |  |  |
| Antibiotic (yes vs. no) | 1.16 (0.54–2.50) | 0.691 |  |  | 1.79 (1.11–2.89) | 0.017 | 1.53 (0.92–2.54) | 0.102 |
| Proton pump inhibitor (yes vs. no) | 1.10 (0.67–1.82) | 0.706 |  |  | 1.14 (0.81–1.60) | 0.464 |  |  |
| Histamine-2-receptor antagonist (yes vs. no) | 3.33 (0.79–14.3) | 0.101 |  |  | 1.83 (1.02–3.28) | 0.042 | 1.55 (0.83–2.88) | 0.170 |
| Glucocorticoid (yes vs. no) | 3.23 (0.78–14.29) | 0.108 |  |  | 1.74 (0.96–3.13) | 0.067 | 1.41 (0.75–2.63) | 0.287 |
| Nonsteroidal anti-inflammatory drug (yes vs. no) | 1.19 (0.60–2.38) | 0.613 |  |  | 1.19 (0.76–1.86) | 0.459 |  |  |
| Calcium channel blocker (yes vs. no) | 0.00 (0.00–Inf) | 0.979 |  |  | 1.61 (0.87–2.96) | 0.128 |  |  |
| Insulin (yes vs. no) | 1.96 (0.46–8.33) | 0.368 |  |  | 1.16 (0.54–2.51) | 0.698 |  |  |
| Opioid (yes vs. no) | 1.18 (0.59–2.33) | 0.639 |  |  | 1.03 (0.65–1.63) | 0.917 |  |  |

Abbreviations: AFP, α-fetoprotein; BCLC, Barcelona Clinic Liver Cancer; ECOG PS, Eastern Cooperative Oncology Group performance status; HBV, hepatitis B virus; OR, odds ratio.

# Figure S1. Time to progression of hepatocellular carcinoma patients with or without concomitant use of commonly prescribed medications.


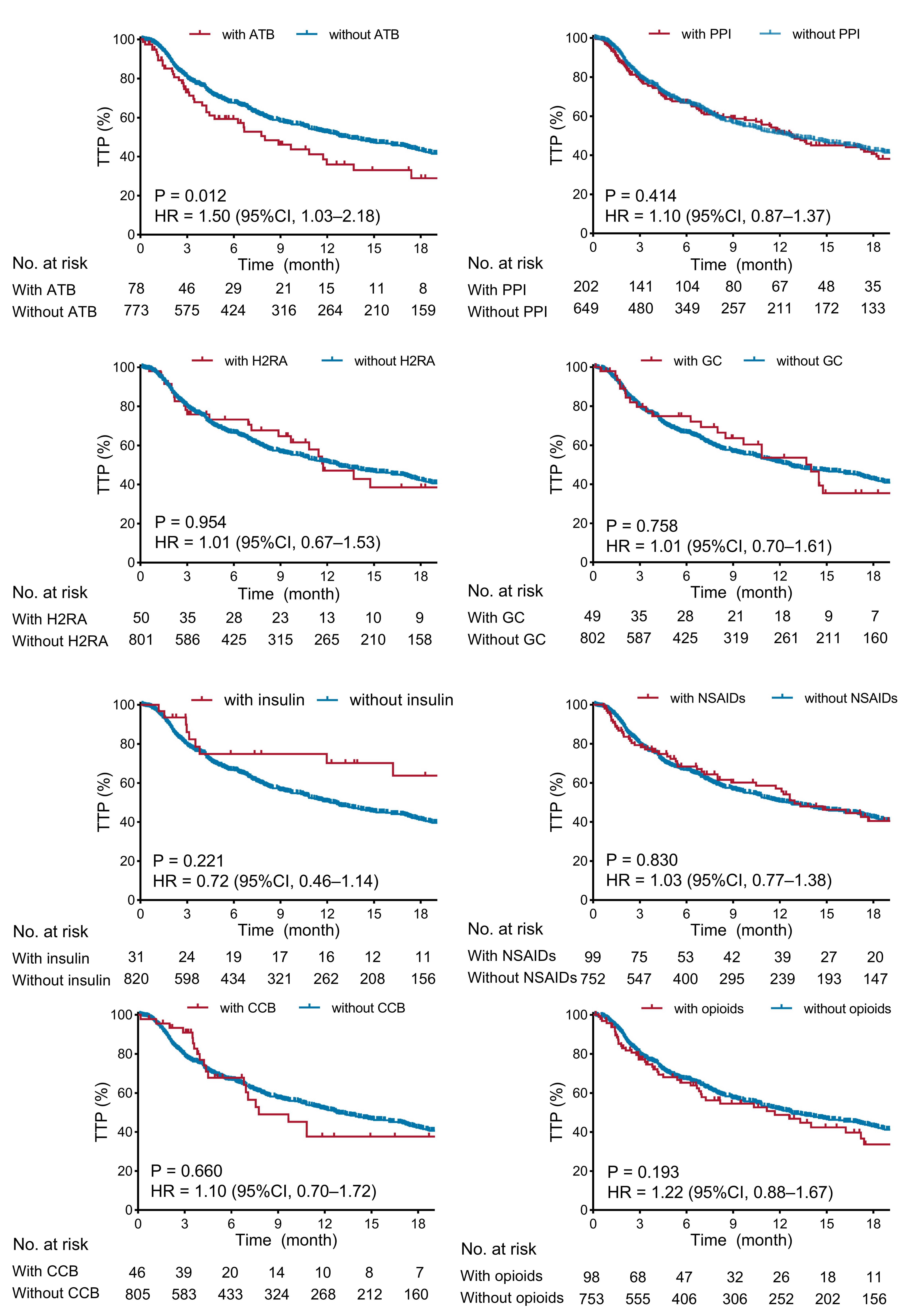


Kaplan-Meier curves showed that time to progression (TTP) was significantly decreased in patients with concomitant antibiotic use compared to those without antibiotic exposure (A), while the TTP was similar in patients with or without PPI (12.8 months vs. 12.6 months; P = 0.414) (B), H2RA (11.7 months vs. 12.6 months; P = 0.954) (C), GC (14.0 months vs. 12.6 months; P = 0.758) (D), insulins (21.7 months vs. 12.3 months; P = 0.221) (E), NSAIDs (12.9 months vs. 12.4 months; P = 0.830) (F), CCB (7.8 months vs. 12.7 months; P = 0.660) (G), and opioids (11.7 months vs. 12.7 months; P = 0.193) (H). ATB, antibiotics; CCB, calcium channel blocker; CI, confidence interval; GC, glucocorticoid; H2RA, histamine-2-receptor antagonist; HR, hazard ratio; NSAIDs, nonsteroidal anti-inflammatory drug; PPI, proton pump inhibitor; TTP, time to progression.

# Figure S2. Overall tumor response regarding concomitant medication according to the Response Evaluation Criteria In Solid Tumors (RECIST) 1.1 criteria.


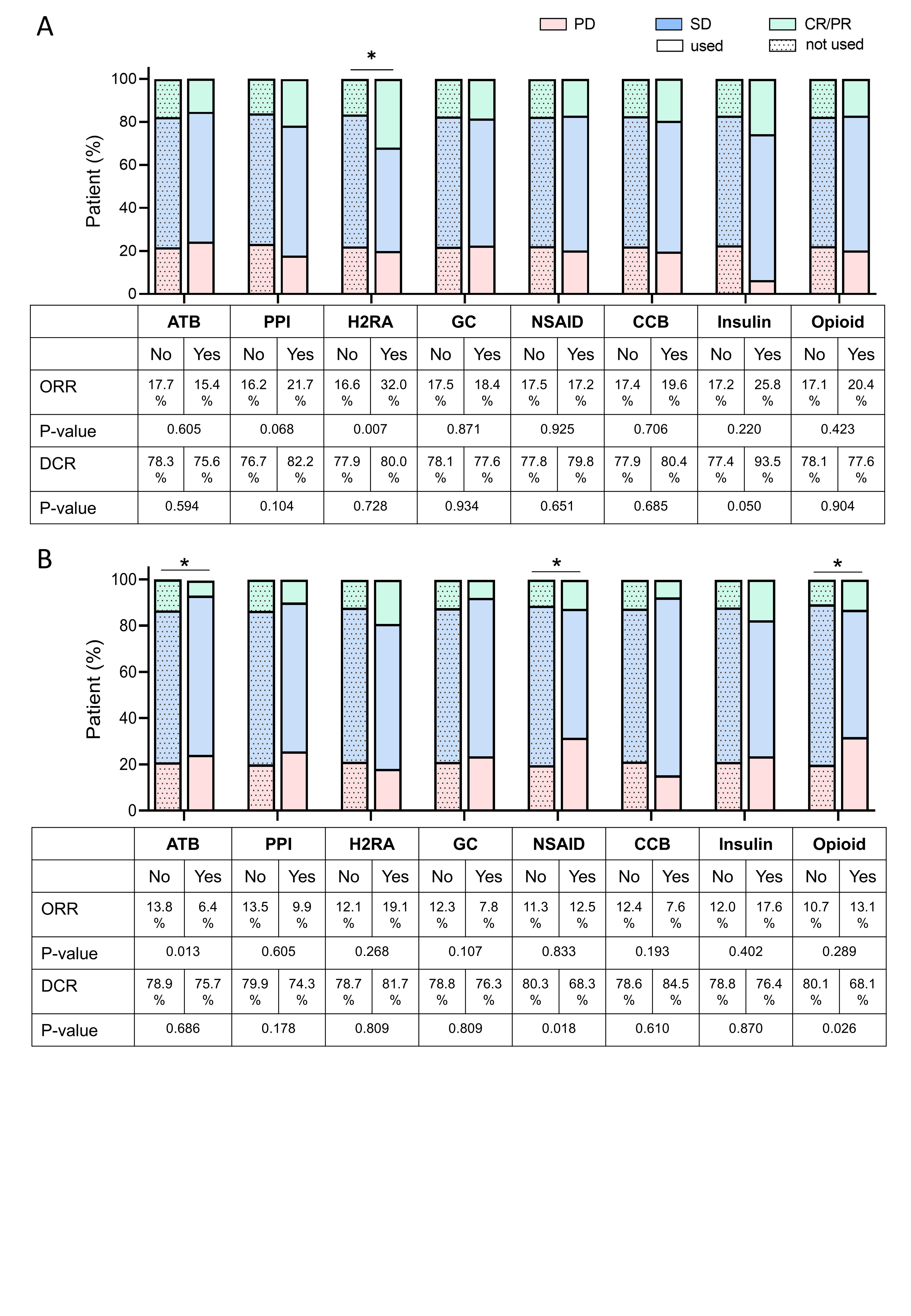


The column chart and table detailed profile of the overall tumor response based on the best of response (A) or the first imaging follow-up (B). ATB, antibiotic; CCB, calcium channel blocker; GC, glucocorticoid; H2RA, histamine-2-receptor antagonist; NSAIDs, nonsteroidal anti-inflammatory drug; PPI, proton pump inhibitor; * P < 0.05.

# Figure S3. Subgroup analyses on survival outcomes in hepatocellular carcinoma patients with concomitant antibiotic use.


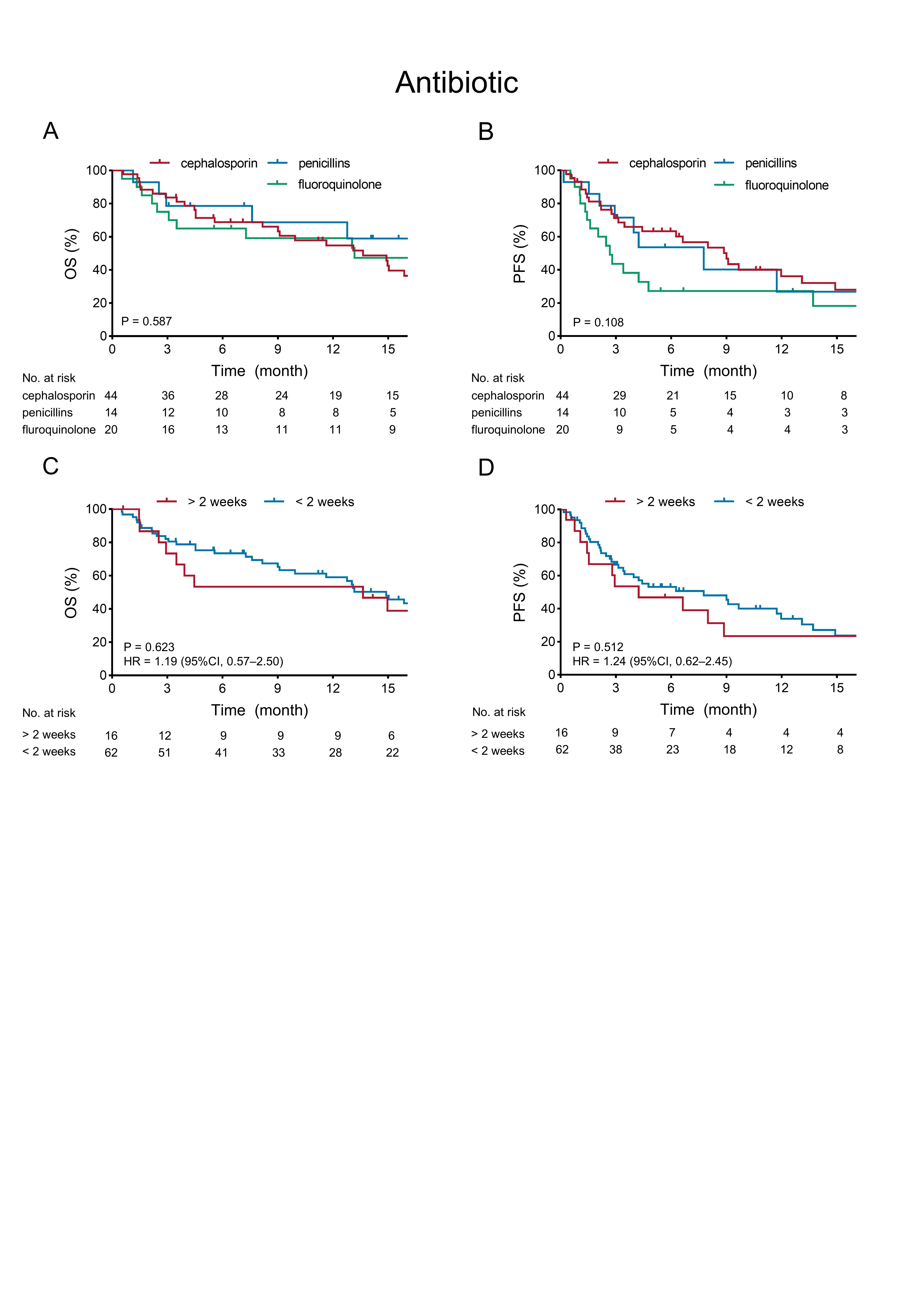


Kaplan-Meier curves showed similar overall survival and progression-free survival in groups receiving different types of antibiotics (OS: P = 0.587; PFS: P = 0.108) (A, B) and in group treated with antibiotics for > 2 weeks compared to that with antibiotics for < 2 weeks (OS: 13.6 months vs. 14.9 months; P = 0.623; PFS: 4.2 months vs. 7.8 months; P = 0.512) (C, D). CI, confidence interval; HR, hazard ratio; PFS, progression-free survival; OS, overall survival.

# Figure S4. Univariable and multivariable Cox regression analyses for time to progression of hepatocellular carcinoma patients treated with immune-based therapy.


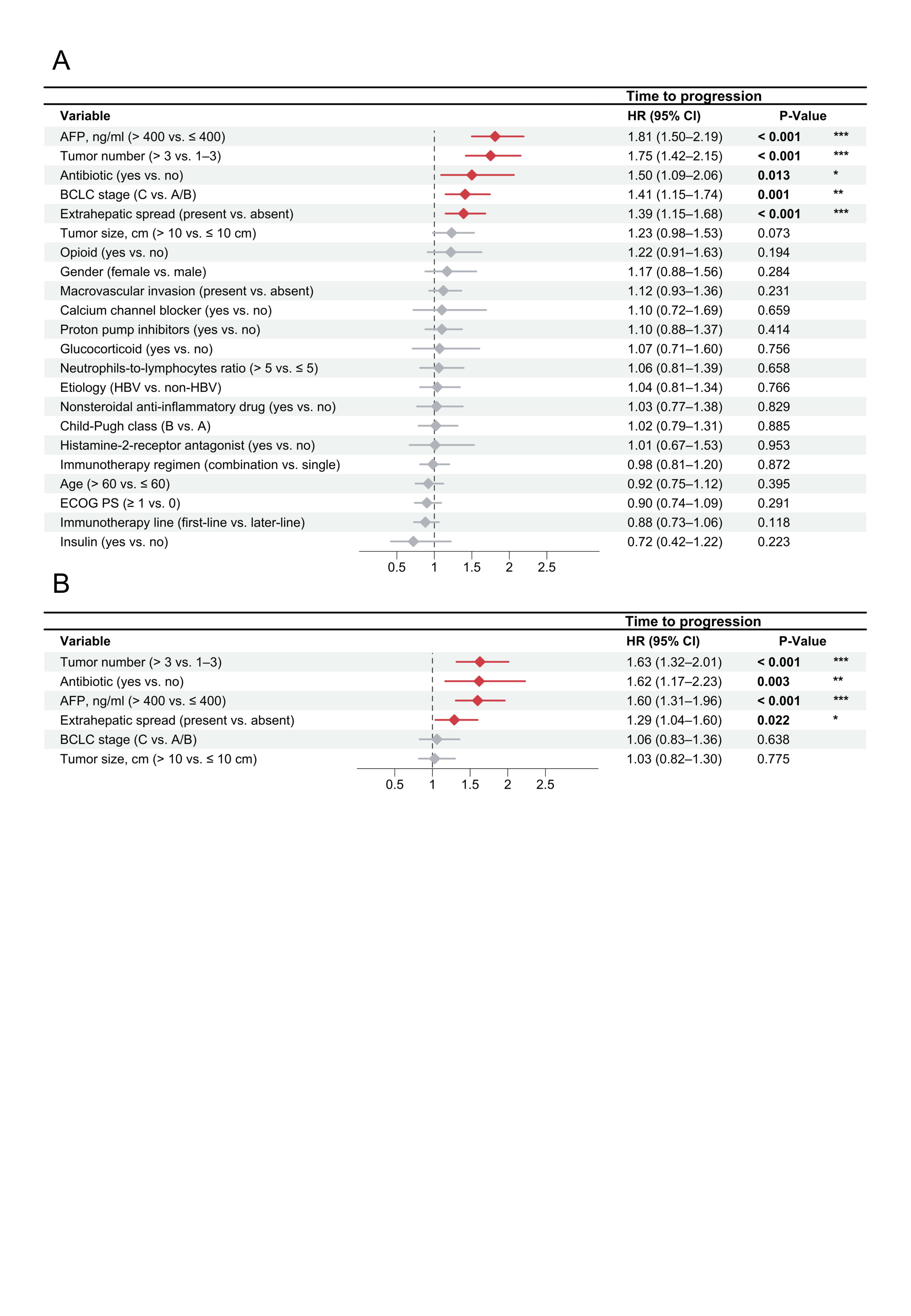


The association between clinical variables and time to progression was evaluated through univariable Cox regression analyses (A) and multivariable Cox regression analyses (B). Abbreviation: AFP, α-fetoprotein; BCLC, Barcelona Clinic Liver Cancer; ECOG PS, Eastern Cooperative Oncology Group performance status; HBV, hepatitis B virus; OR, hazard ratio

# Figure S5. Subgroup analyses on survival outcomes in hepatocellular carcinoma patients with concomitant proton pump inhibitor use.


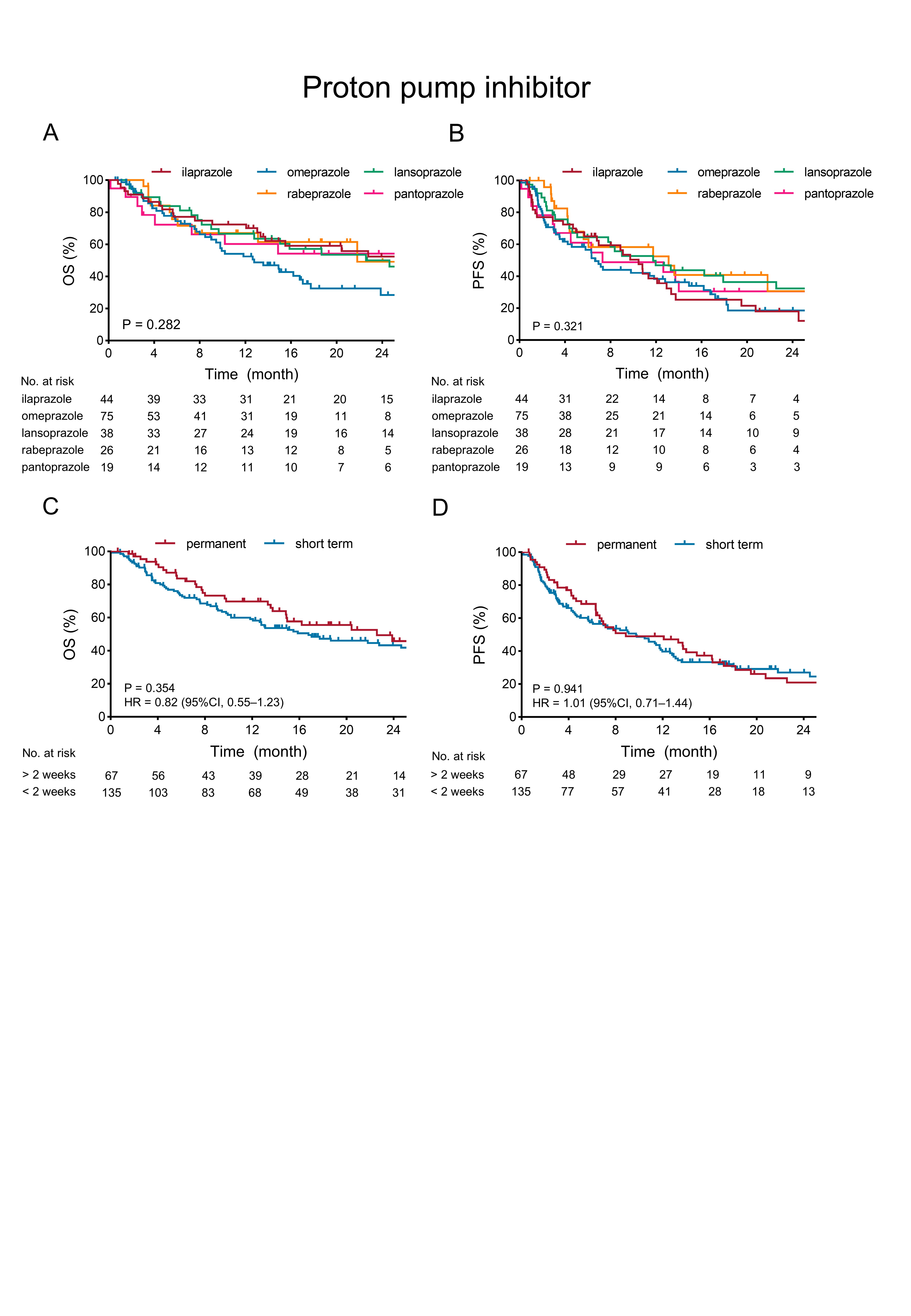


Kaplan-Meier curves showed similar overall survival and progression-free survival in groups receiving different types of PPIs (OS: P = 0.282; PFS: P = 0.321) (A, B) and in group treated with permanent PPIs compared to that with short-term PPIs (OS: 22.6 months vs. 16.8 months; P = 0.354; PFS: 8.9 months vs. 9.7 months; P = 0.941) (C, D). CI, confidence interval; HR, hazard ratio; PFS, progression-free survival; OS, overall survival.

# Figure S6. Subgroup analyses on survival outcomes in hepatocellular carcinoma patients with concomitant glucocorticoid use.


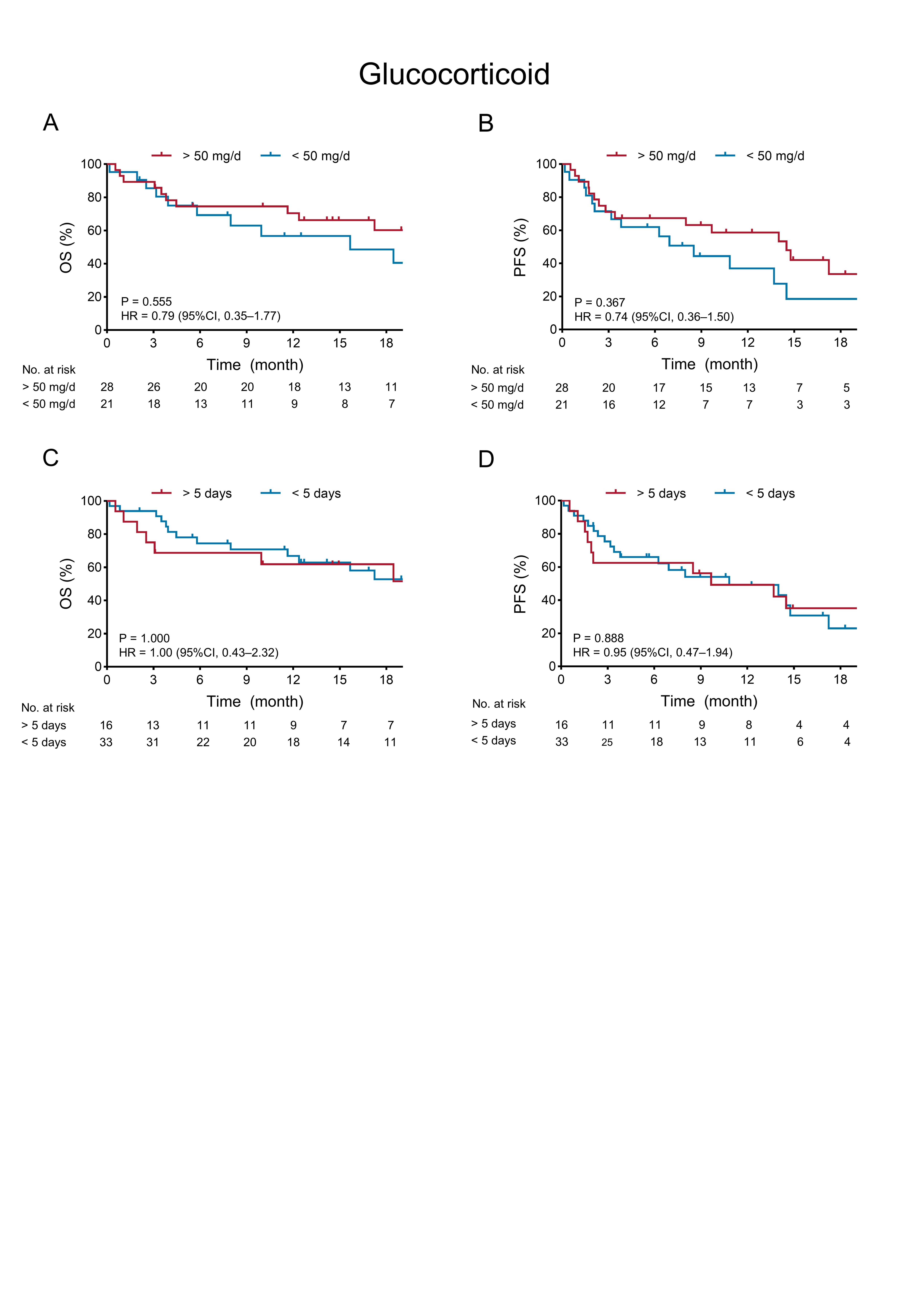


Kaplan-Meier curves showed similar overall survival and progression-free survival in group treated with glucocorticoid for > 50 mg/d compared to that with glucocorticoid for < 50 mg/d (OS: 22.6 months vs. 15.7 months; P = 0.555; PFS: 14.5 months vs. 8.5 months; P = 0.367) (A, B) and in groups treated with glucocorticoid for > 5 days compared to that with glucocorticoid for < 5 days (OS: 22.6 months vs. 20.3 months; P = 1.000; PFS: 9.7 months vs. 10.8 months; P = 0.888) (C, D). CI, confidence interval; HR, hazard ratio; PFS, progression-free survival; OS, overall survival.

# Figure S7. 30-day landmark analyses of survival outcomes of hepatocellular carcinoma patients receiving glucocorticoids based on different indications.


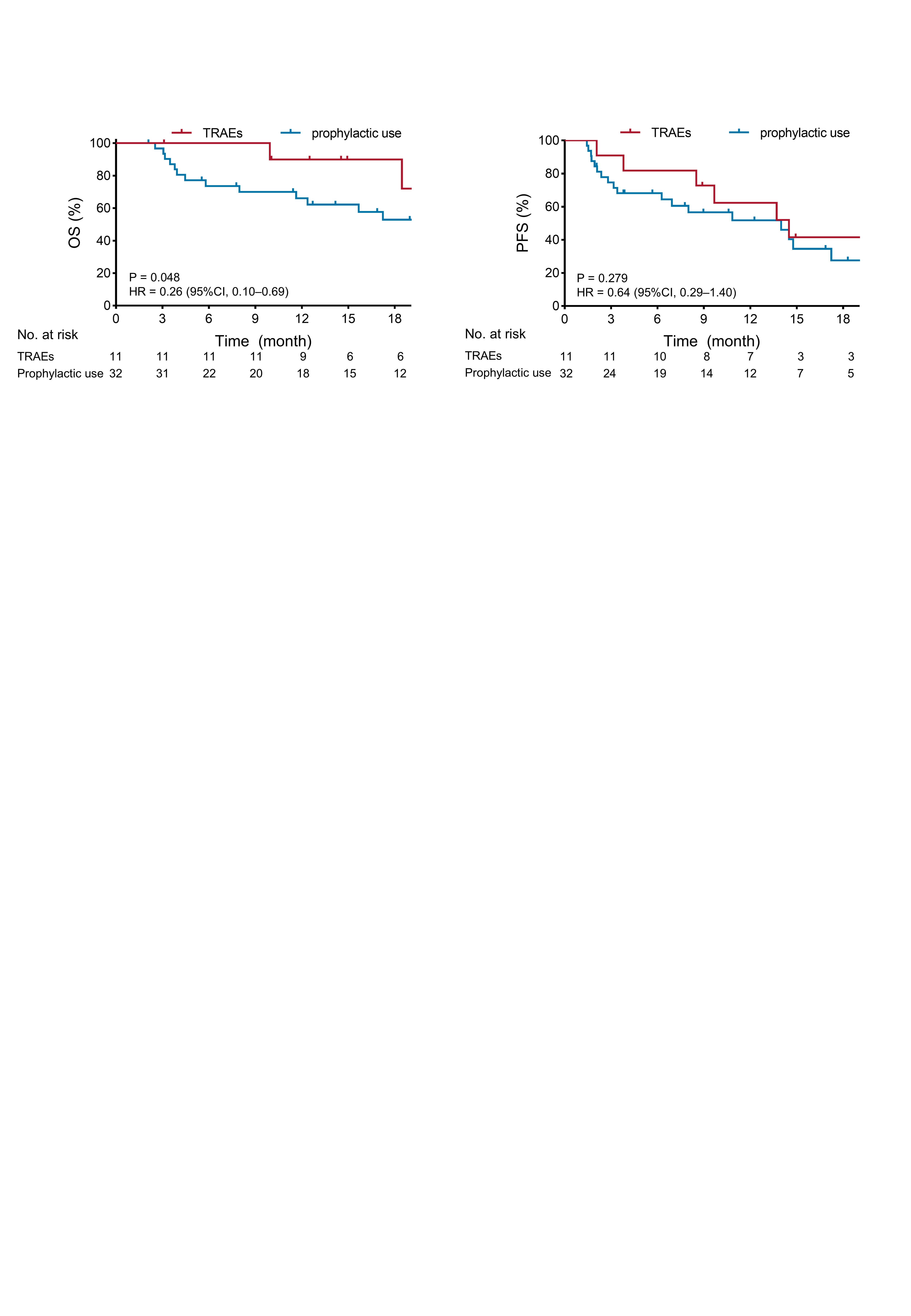


Kaplan-Meier curves after a 30-day landmark selection showed that patients receiving glucocorticoids for early TRAE management had longer OS than those receiving prophylactic glucocorticoids, while PFS did not differ between the two groups. CI, confidence interval; HR, hazard ratio; OS, overall survival; PFS, progression-free survival.

# Figure S8. Overall survival of hepatocellular carcinoma patients with or without concomitant use of other commonly prescribed medications.


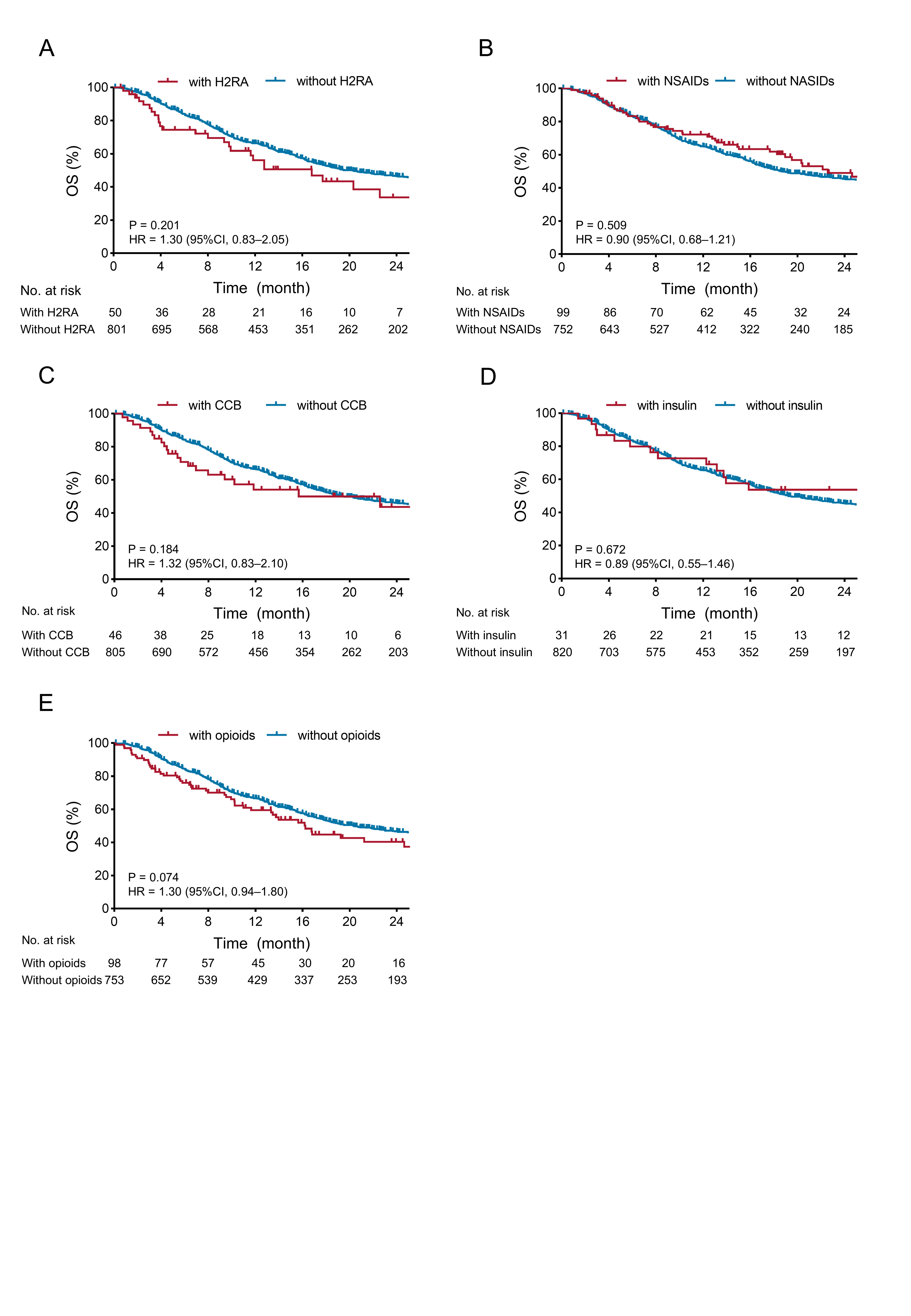


Kaplan-Meier curves showed the overall survival was comparable in patients with or without H2RAs (16.8 months vs. 20.3 months; P = 0.201) (A), NSAIDs (22.6 months vs. 18.7 months; P = 0.509) (B), CCBs (15.7 months vs. 19.4 months; P = 0.184) (C), insulins (26.0 months vs. 19.2 months; P = 0.672) (D), and opioids (16.2 months vs. 20.3 months; P = 0.074) (E). CCB, calcium channel blocker; CI, confidence interval; H2RA, histamine-2-receptor antagonist; HR, hazard ratio; NSAIDs, nonsteroidal anti-inflammatory drug; OS, overall survival.

# Figure S9. Progression-free survival of hepatocellular carcinoma patients with or without concomitant use of other commonly prescribed medications.


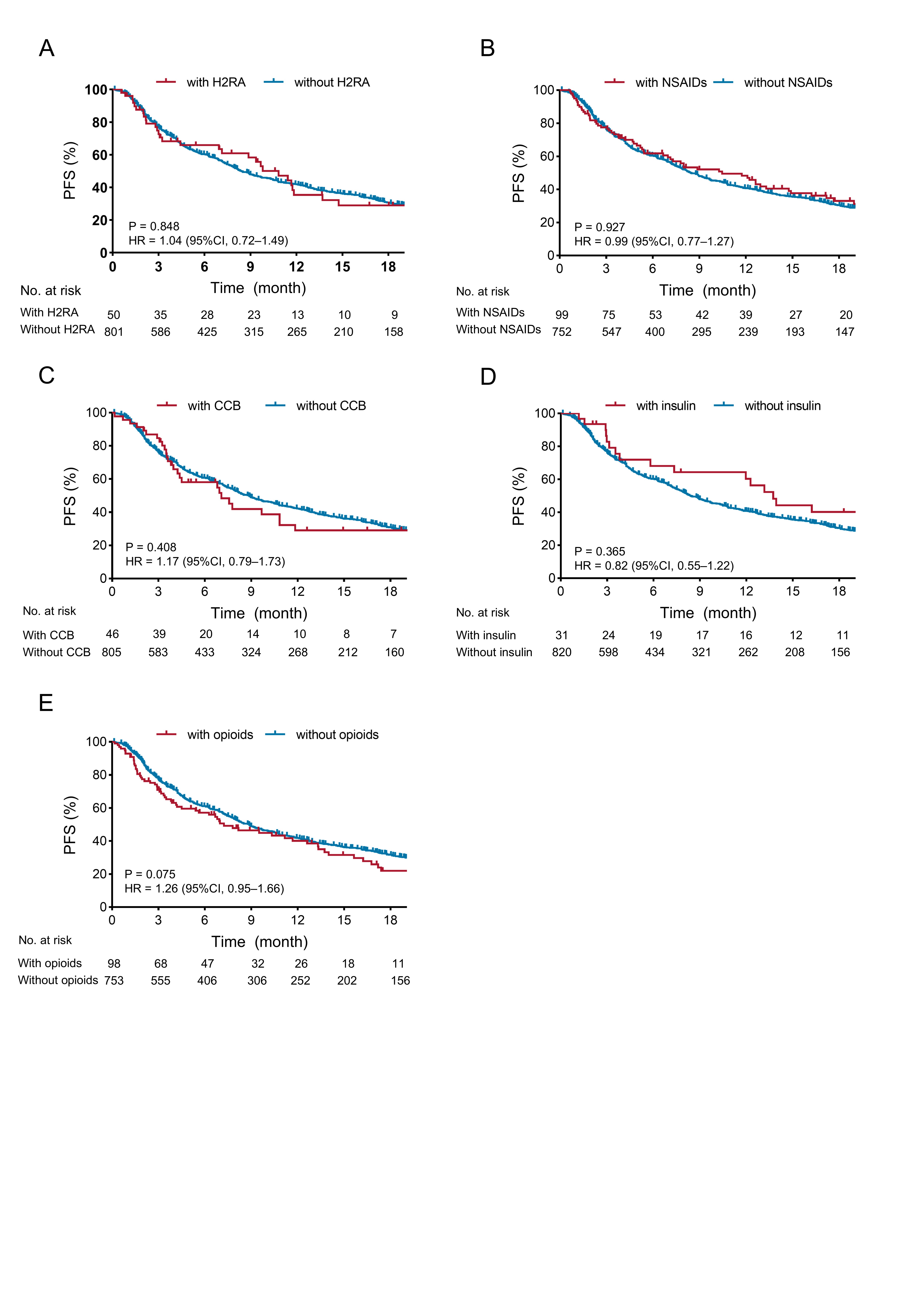


Kaplan-Meier curves showed the progression-free survival was comparable in patients with or without H2RAs (10.8 months vs. 8.4 months; P = 0.848) (A), NSAIDs (10.5 months vs. 8.5 months; P = 0.927) (B) CCBs (7.1 months vs. 8.9 months; P = 0.408) (C); insulins (13.7 months vs. 8.4 months; P = 0.365) (D); and opioids (7.2 months vs. 8.9 months; P = 0.075) (E). CCB, calcium channel blocker; CI, confidence interval; H2RA, histamine-2-receptor antagonist; HR, hazard ratio; NSAIDs, nonsteroidal anti-inflammatory drug; PFS, progression-free survival.

# Figure S10. Subgroup analyses on survival outcomes in patients based on the different number of concomitant medications.


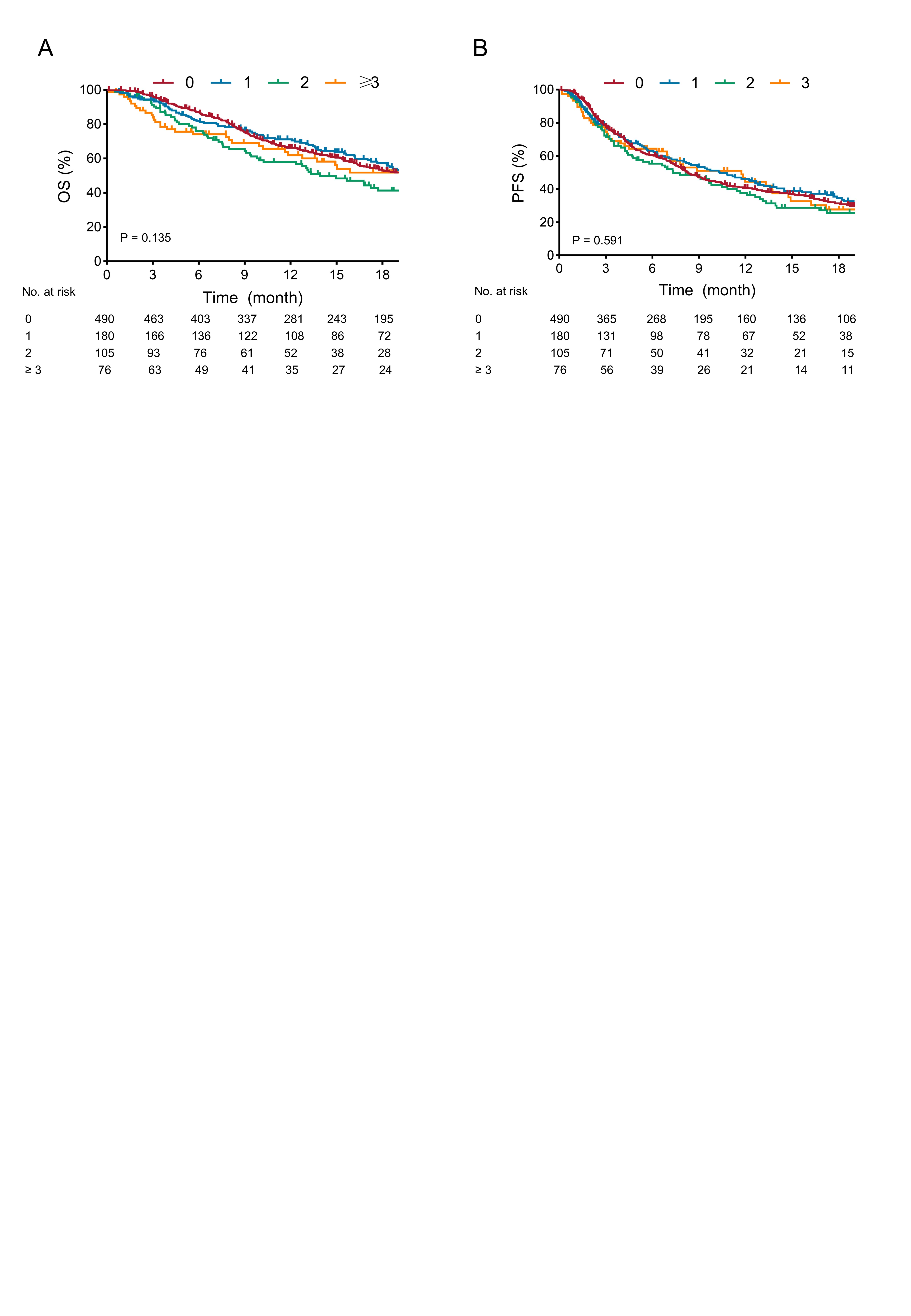


Kaplan-Meier curves showed the OS and PFS were comparable in patients receiving different numbers of concomitant medications (OS: P = 0.135; PFS: P = 0.591) (A, B). CI, confidence interval; HR, hazard ratio; PFS, progression-free survival; OS, overall survival.

# Figure S11. Subgroup analyses on survival outcomes in hepatocellular carcinoma patients based on different concomitant medication combinations.


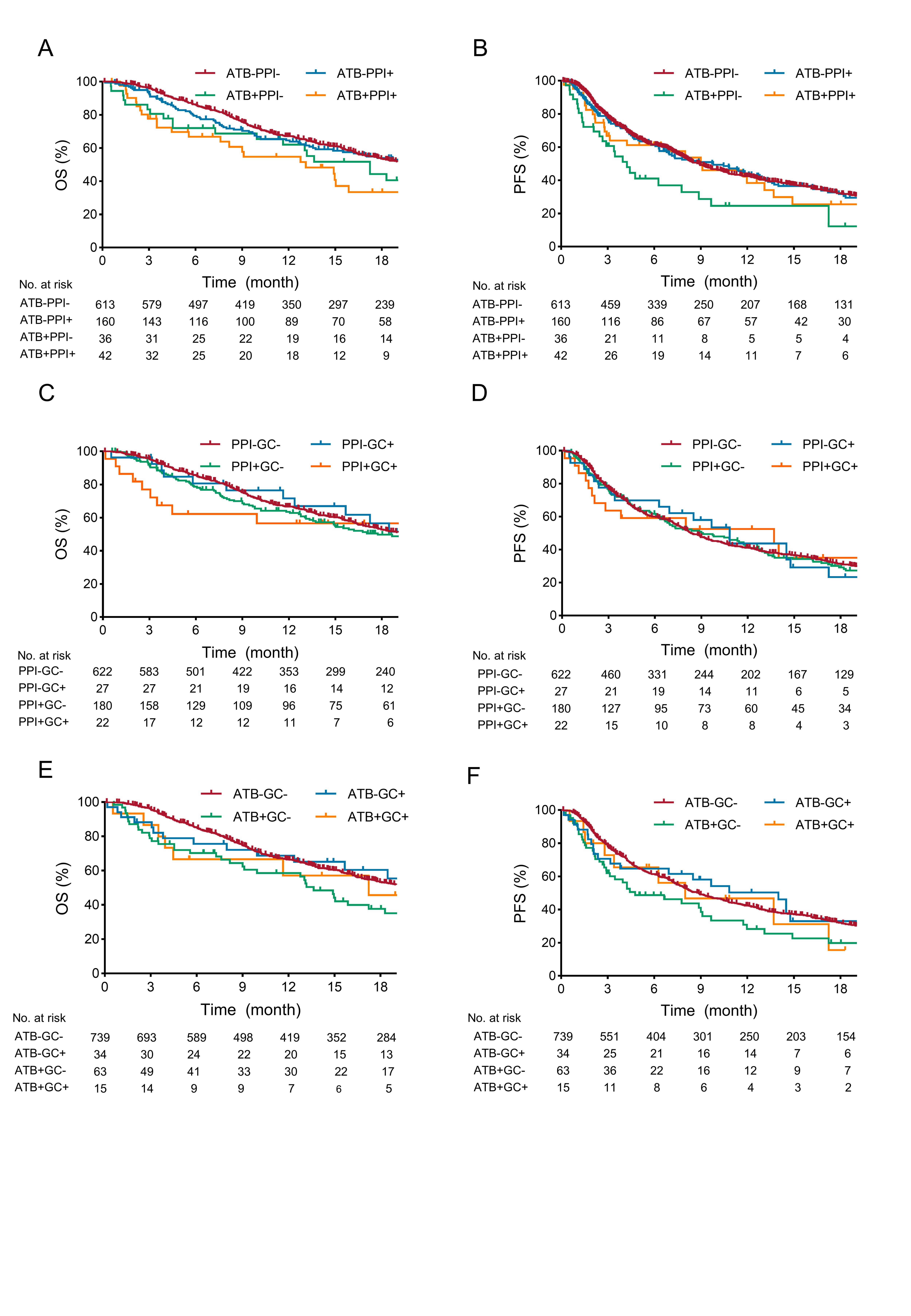


Kaplan-Meier curves showed the overall survival and progression-free survival did not differ in patients with antibiotics plus PPIs (A, B), PPIs plus glucocorticoids (C, D), or antibiotics plus glucocorticoids compared to those with single agent use or no use (E, F). ATB, antibiotic; CI, confidence interval; GC, glucocorticoid; HR, hazard ratio; PFS, progression-free survival; PPI, proton pump inhibitor; OS, overall survival.
